# The backbone network of dynamic functional connectivity

**DOI:** 10.1162/netn_a_00209

**Published:** 2021-11-30

**Authors:** Nima Asadi, Ingrid R. Olson, Zoran Obradovic

**Affiliations:** Department of Computer and Information Sciences, College of Science and Technology, Temple University, Philadelphia, PA, USA; Department of Psychology, College of Liberal Arts, Temple University, Philadelphia, PA, USA; Decision Neuroscience, College of Liberal Arts, Temple University, Philadelphia, PA, USA; Department of Computer and Information Sciences, College of Science and Technology, Temple University, Philadelphia, PA, USA

**Keywords:** Dynamic functional connectivity, Backbone network, Null model, Optimization, Autism spectrum disorder

## Abstract

Temporal networks have become increasingly pervasive in many real-world applications, including the functional connectivity analysis of spatially separated regions of the brain. A major challenge in analysis of such networks is the identification of noise confounds, which introduce temporal ties that are nonessential, or links that are formed by chance due to local properties of the nodes. Several approaches have been suggested in the past for static networks or temporal networks with binary weights for extracting significant ties whose likelihood cannot be reduced to the local properties of the nodes. In this work, we propose a data-driven procedure to reveal the irreducible ties in dynamic functional connectivity of resting-state fMRI data with continuous weights. This framework includes a null model that estimates the latent characteristics of the distributions of temporal links through optimization, followed by a statistical test to filter the links whose formation can be reduced to the activities and local properties of their interacting nodes. We demonstrate the benefits of this approach by applying it to a resting-state fMRI dataset, and provide further discussion on various aspects and advantages of it.

## Author Summary

In this work we propose an optimization-based null model to infer the significant ties, meaning the links that cannot be reduced to the local strengths and properties of the nodes, from the dynamic functional connectivity network. We asses multiple aspects of this approach and demonstrate that it is adaptable to most temporal segmentation methods. We demonstrate that this approach provides several advantages such as taking into account the global information of the network. We also compare the proposed model with several commonly applied null models empirically and theoretically.

## INTRODUCTION

Dynamic functional connectivity (dFC) has been widely used to analyze temporal associations among separate regions of the brain as well as the correlation between functional patterns of connectivity and cognitive abilities (Allen et al., 2014; Jones et al., 2012; Van Dijk et al., 2010; von der Malsburg, Phillps, & Singer, 2010). In order to identify coactivation patterns of dFC over the period of experiment, a temporal segmentation (such as sliding window) is commonly applied on the time courses of BOLD activation of brain regions to divide them into consecutive temporal windows (Allen et al., 2014; Hutchison et al., 2013; Smith et al., 2012). Then, the connectivity between separate regions is measured to generate one graph adjacency matrix per each temporal window (Damaraju et al., 2014). Building on this core framework, several enhancements have been proposed in the past years, such as different temporal segmentation approaches, to increase the power and precision of dFC analysis (Chang & Glover, 2010; Heitmann & Breakspear, 2018; Hindriks et al., 2016; Kiviniemi et al., 2011).

However, a major challenge in analysis of dynamic functional connectivity is to distinguish and address the existing noise confounds in the data, which influence the brain connectivity measures and the structure of the dFC network (Birn, Smith, Jones, & Bandettini, 2008; Chang & Glover, 2009; Kalthoff, Seehafer, Po, Wiedermann, & Hoehn, 2011; Shmueli et al., 2007). This issue especially intensifies with the increase in spatial resolution of the analysis as well as in resting-state fMRI data (Birn, 2012; Hallquist, Hwang, & Luna, 2013; Kalthoff et al., 2011). There are several possible sources of noise in resting-state fMRI data, including displacements, even as small as a millimeter or less, which could add random noise to the generated time series, and therefore decrease the statistical power in resting-state functional connectivity (rsFC) analysis (Van Dijk, Sabuncu, & Buckner, 2012). Even more challenging, it can result in false positive or negative activation if the displacements are correlated with the stimuli (Lydon-Staley, Ciric, Satterthwaite, & Bassett, 2019; Patanaik et al., 2018; Savva, Kassinopoulos, Smyrnis, Matsopoulos, & Mitsis, 2020). Cardiovascular and respiratory signals are also widely identified as a source of noise, causing synchronized fluctuations in MRI signal (Glover & Lee, 1995).

Because of these challenges, neuroscientists often face the concern of analytical models being noise-induced (Choe et al., 2017; Gorgolewski, Storkey, Bastin, Whittle, & Pernet, 2013; Murphy, Birn, & Bandettini, 2013). A number of correction techniques have been suggested in the past to reduce the influence of these confounds, including modeling fMRI signal variations using independent measures of the cardiac and respiratory signal variations (Behzadi, Restom, Liau, & Liu, 2007; Bollmann et al., 2017; Bright & Murphy, 2017; Murphy et al., 2013). However, the effect of various sources of noise on the dynamic connectivity of fMRI data is yet to be addressed through a data-driven and systematic framework (Beall & Lowe, 2007; Kundu, Inati, Evans, Luh, & Bandettini, 2012).

Moreover, temporal ties that can be reduced to node properties can exist between nodes because of the nature of the data itself. Highly active regions could in principle form a larger number of trivial ties with other regions, and reciprocally, the information of ties that regions with lower activity form can be lost in common analytical procedures (Gemmetto, Cardillo, & Garlaschelli, 2017; Kobayashi, Takaguchi, & Barrat, 2019). In general, if the network representation of a real-world system can be inferred based on local properties of the nodes, such as their activity level or degree, the true interaction and functional homologies between the nodes can not be detected (Gemmetto et al., 2017).

Therefore, the objective of this work is to put forward a data-driven approach to distinguish the significant ties that construct the functional connectivity of the brain from ties that are the result of random observational errors or chance. The latter group of temporal links are known as reducible ties, whereby they can be fully attributed to intrinsic node-specific features such as degree or strength of their link weights. On the other hand, the temporal ties that are incompatible with the null hypothesis of links being produced at random are known as irreducible or significant ties, and the network of such significant ties is known as the backbone network. Therefore, the goal of this study is to develop a data-driven framework to infer the two-dimensional backbone network from the multilayer network of dynamic functional connectivity.

Multiple approaches have been proposed to extract the significant ties in a network through statistical means, most of which target static networks (Alvarez-Hamelin, Dall’Asta, Barrat, & Vespignani, 2005; Casiraghi, Nanumyan, Scholtes, & Schweitzer, 2017; Gemmetto et al., 2017; Kobayashi et al., 2019; Ma, Ma, Zhang, & Wang, 2016; Nadini, Bongiorno, Rizzo, & Porfiri, 2020; Serrano, Boguná, & Vespignani, 2009; Tumminello, Micciche, Lillo, Piilo, & Mantegna, 2011; Yan, Jeub, Flammini, Radicchi, & Fortunato, 2018). Across these approaches, a key step towards inferring the backbone network is the formulation of a reliable null model to characterize the reducible fraction of the temporal interactions, and to steer the process of filtering that fraction of network links. Several null models have been suggested in the literature whose focus is on static networks, spanning from basic weight thresholding of multilayer networks to more advanced techniques (Cimini et al., 2019; Kobayashi et al., 2019; Li et al., 2014; Tumminello et al., 2011).

One of the main disadvantages with weight thresholding approaches is that they commonly fail to control for the difference in intrinsic attributes of the nodes, thus they favor highly active nodes or nodes with other strong local properties, which can potentially have a large number of reducible links. A number of null models have been used to evaluate the statistical significance of dFC based on generating null data using randomization frameworks. Two main approaches of this type include autoregressive randomization (ARR) and phase randomization (PR) (Allen et al., 2014; Chang & Glover, 2010; Handwerker, Roopchansingh, Gonzalez-Castillo, & Bandettini, 2012; Zalesky, Fornito, Cocchi, Gollo, & Breakspear, 2014). In this category of time series–based approaches, null hypothesis testing is then applied by comparing statistics from the original data against those from the generated null data. A backbone approach named significant tie filtering (ST filter) for dynamic networks was proposed by Kobayashi et al. (2019), based on a network modeling concept named activity-driven network (ADN) where the individual propensity of generating connections over time is determined by a latent nodal parameter commonly known as activity, and the probability of creating a link at a specific time instant between two nodes is the product of the individual latent activities of interacting nodes (Perra, Gonçalves, Pastor-Satorras, & Vespignani, 2012; Starnini & Pastor-Satorras, 2014; Zino, Rizzo, & Porfiri, 2017). Because of their analytical flexibility and interpretability, activity-driven network models have gained popularity in explaining features of real networks in various areas of research (Liu, Perra, Karsai, & Vespignani, 2014; Rizzo, Frasca, & Porfiri, 2014; Zino et al., 2017). However, in the mentioned studies, a binomial or Poisson distribution is considered for the temporal connections over time, which limits the approach to unweighted networks (Kobayashi et al., 2019; Nadini et al., 2020), whereas many relational networks based on real data, including various types of fMRI-based networks, have continuous weights containing significant information regarding the interactions between the nodes as well as the local and global properties of the network. Therefore, inspired by the work of Kobayashi et al. (2019), we propose an approach for extracting the significant ties for temporal networks with continuous weights that meet the characteristics of normality and independence of temporal ties, which are discussed in the Methodology section. We demonstrate that this methodology controls for intrinsic local node attributes, with a null model that takes into account not only the global structure of the network, but also the temporal variations of the dynamic connectivity links. In the next section we explain the proposed approach in detail, followed by the experimental results on a real dataset of resting-state fMRI. We then present an analysis of the results and discuss the advantages and shortcomings of our approach.

## METHODOLOGY

In this section, we outline the methodological framework for identifying the significant links from the networks of dynamic resting-state functional connectivity. A key step towards extracting the backbone network is the formulation of a valid and robust null model. For the sake of simplicity, we name our proposed approach the weighted backbone network (WBN). A null model assumes that all connections are formed randomly, meaning that the probability of an interaction between two nodes at a specific time window and the weight of interactions between them could be explained by chance (Gemmetto et al., 2017; Kobayashi et al., 2019; Nadini et al., 2020). The objective of inferring the backbone network is thus to detect links that are not compatible with the null hypothesis, meaning that their formation or strength is not driven by chance.

The null model that we present can be interpreted as a temporal fitness model, which is characterized by latent parameters that shape its distribution. In this vein, the first step is to estimate these parameters that are not directly observed from the data. For this purpose, we use a maximum likelihood estimation (MLE) approach that exploits the global and temporal information of the network of dynamic connectivity. We discuss the details of this methodology in the next section.

### Estimation of Latent Distribution Variables

We consider a dynamic network of *N* nodes with links evolving over *τ* observation windows of size Δ such that *t* = 1, …, *τ*. At each time step *t*, a weighted undirected network is formed whose adjacency matrix *A*_*t*_ stochastically varies in time, and the weights of temporal links (links that are formed at time step *t*) between each pair of nodes *i* and *j* form a Gaussian distribution over the *τ* time steps. Normality of the distribution of weights between each pair of nodes over time *τ* is concluded based on the central limit theorem and the assumption that the distribution of temporal weights has a finite variance (Dudley, 1978, 2014; Haller & Bartsch, 2009; Smith, 2012). Moreover, an empirical assessment of normality of the distribution of temporal weights on a real dataset of resting-state fMRI is provided in the Results section.

We define a temporal null model in which each node *i* is assigned two intrinsic variables *a*_*i*_, *b*_*i*_ ∈ (0, 1], that rule the probability of mean *μ* and standard deviation *σ* of the temporal distribution of its interactions with other nodes over *τ* time steps, such that

$$\begin{array}{l} \mu_{i,j}=a_{i}\times a_{j},\\ \sigma_{i,j}=b_{i}\times b_{j}. \end{array}$$ (1)

Therefore, each parameter of the distributions of temporal ties between each pair of node *i* and *j* is the realization of a Bernoulli variable. The null model thus lays out a baseline for the expected mean and standard deviation of the distribution of interactions between two nodes over *τ* time given their intrinsic variables, if interacting nodes are selected at random at each time step.

To uncover significant links with regards to the null model described above, we proceed in two steps. First, given a set of weighted undirected temporal networks with *N* nodes, we estimate the intrinsic variables a* = ($a_{1}^{*}$, …, $a_{N}^{*}$) and b* = ($b_{1}^{*}$, …, $b_{N}^{*}$) by calculating the maximum likelihood estimation of the set of parameters for each node. For this purpose, we consider the joint probability function over *τ* time intervals and edge weights *w* ∈ ($w_{i,j}^{t}$; *i*, *j* ∈ 1, …, *N*; *t* ∈ 1, …, *τ*) for the entire temporal connections of the network. Therefore we have the following:

$$f\left(w_{i,j}^{t}|\mu_{i,j},\sigma_{i,j}\right)=\prod_{i,j,i\neq j}\prod_{t=1}^{\tau }\frac{1}{\sigma_{i,j}\sqrt{2\pi }}\;e^{-{\left(w_{i,j}^{t}-\mu_{i,j}\right)}^{2}/2\sigma_{i,j}^{2}},$$ (2)

where *μ*_*i*,*j*_ and *σ*_*i*,*j*_ denote the mean and standard deviation of the distribution of temporal edges between nodes *i* and *j* observed over *τ* time steps in the null model.

The log-likelihood function for the empirical data $w_{i,j}^{t}$ (weight of the link between *i* and *j* at time interval *t*) with replacing the values of *μ*_*i*,*j*_ = *a*_*i*_.*a*_*j*_ and *σ*_*i*,*j*_ = *b*_*i*_.*b*_*j*_ will lead to:

$$\log\left(f\left(w_{i,j}^{t}|\mu_{i,j},\sigma_{i,j}\right)\right)=\sum_{i,j,i\neq j}\left[-n\log\left(b_{i}b_{j}\right)-\frac{n}{2}\log2\pi -\sum_{t=1}^{\tau }\frac{1}{2{\left(b_{i}b_{j}\right)}^{2}}{\left(w_{i,j}^{t}-a_{i}a_{j}\right)}^{2}\right]$$ (3)

By differentiating the log-likelihood function with respect to the first parameter, *a*_*i*_, and setting it to zero we have

$$\sum_{j,j\neq i}\left[a_{i}^{*}a_{j}^{*}-\sum_{t=1}^{\tau }\frac{w_{i,j}^{o}}{\tau }\right]=\sum_{j,j\neq i}\left[a_{i}^{*}a_{j}^{*}-w_{i,j}^{o}\right]=0,\forall i=1,\ldots ,N.$$ (4)

Similarly, by differentiating the log-likelihood function with respect to *b*_*i*_ and setting it to 0 we have

$$\sum_{j,j\neq i}\left[-{\left(b_{i}^{*}b_{j}^{*}\right)}^{2}+\sum_{t=1}^{\tau }\frac{{\left(w_{i,j}^{o}-a_{i}^{*}a_{j}^{*}\right)}^{2}}{\tau }\right]=0,\forall i=1,\ldots ,N,$$ (5)

in which the the maximum likelihood estimation of $a_{i}^{*}$ for every node *i* was calculated from Equation 4. Therefore, for a temporal network with *N* nodes, the pair of latent variables *a*_*i*_, *b*_*i*_ for each node *i* can be estimated by solving the system of *N* nonlinear Equations 4 and 5. The system of nonlinear equations can be solved through a standard numerical algorithm such as the Newton method. The initial values of *a*_*i*_ and *b*_*i*_ are calculated by dividing the temporal degree of node *i* averaged over *τ* time steps by the doubled number of total temporal edges as follows:

$$a_{i}=\sum_{j,j\neq i}\sum_{t=1}^{\tau }w_{i,j}/\tau \sqrt{2*\sum_{i<j}w_{i,j}/\tau }.$$ (6)

**Figure 1.**
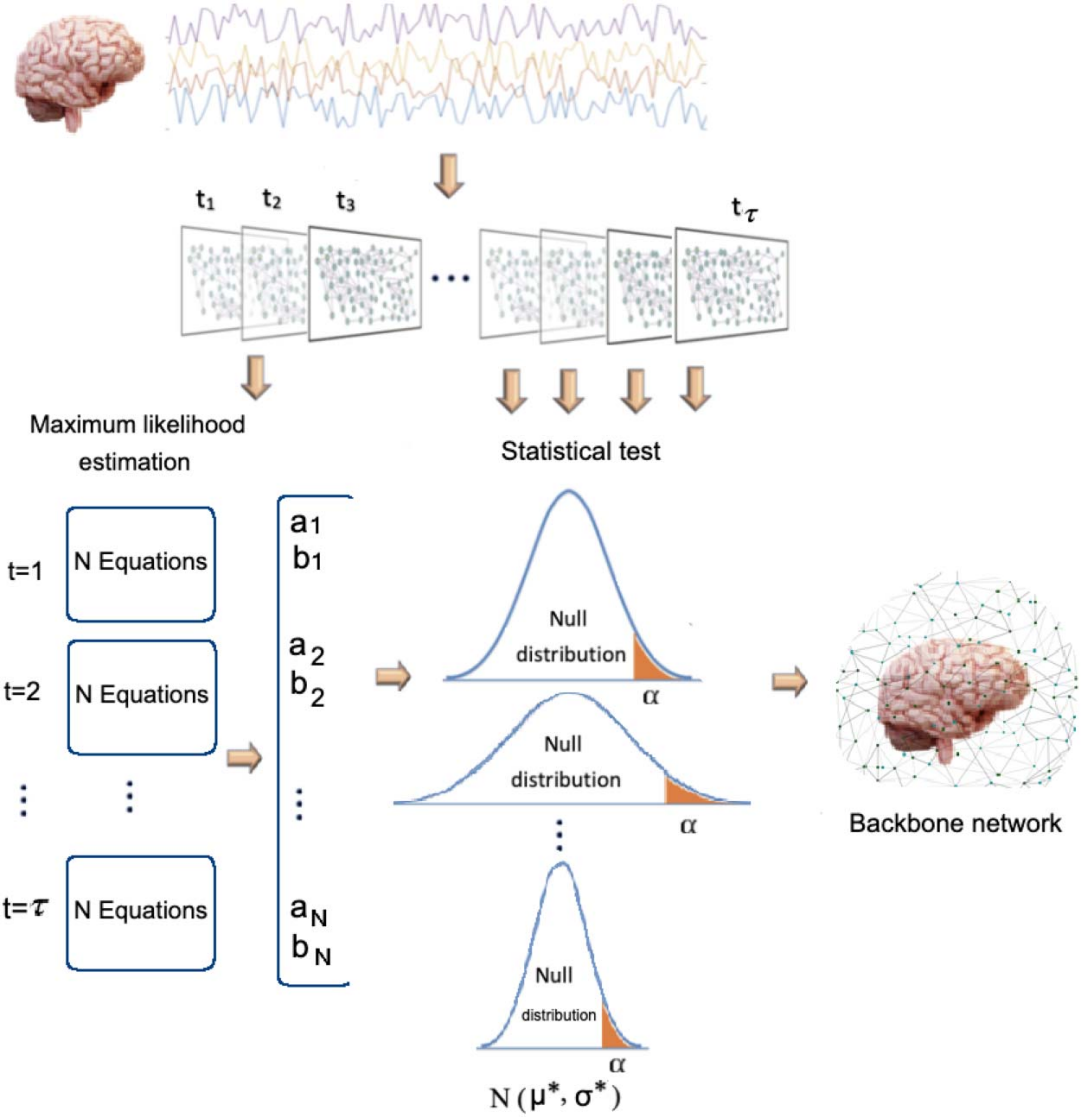
A schema of the backbone network inference procedure.

The general schema of the proposed methodology is provided in Figure 1. Note that the proposed maximum likelihood approach incorporates the global information of the network, as the weights of all temporal links are considered in the system of equations.

### Selection of Significant Ties

After estimating the latent distribution variables for each node, we then compute for each pair of nodes *i* and *j* the probability distribution of their interaction over the *τ* time steps in the null model:

$$g\left(w_{ij}^{t}|\mu_{i,j}^{*},\sigma_{i,j}^{*}\right)=\frac{1}{\sigma_{i,j}^{*}\sqrt{2\pi }}e^{-{\left(w_{ij}^{t}-\mu_{i,j}^{*}\right)}^{2}/2{\sigma_{i,j}^{*}}^{2}},$$ (7)

where $\mu_{i,j}^{*}$ and $\sigma_{i,j}^{*}$ are the mean and standard deviation based on the estimated latent variables *a*_*i*_, *b*_*i*_, *a*_*j*_, *b*_*j*_ through maximum likelihood estimation and Equation 1. In order to determine the reducibility of a temporal link $w_{i,j}^{t}$ between nodes *i* and *j* at time *t*, it is compared against the *c*th percentile (0 ≤ *c* ≤ 100) weight $w_{ij}^{c}$ of the maximum likelihood estimated distribution of temporal links between *i* and *j*. If the empirical value $w_{i,j}^{t}$ is larger than the $w_{ij}^{c}$, then it cannot be explained by the null model at significance level *α* = 1 − $\frac{c}{100}$. Therefore, the link $w_{i,j}^{t}$ is determined to be a significant tie. The significance level *α* is given as an input to the model, providing a systematic adjustable filtering mechanism. The significance threshold *α* can also be assigned with Bonferroni correction, in which it can be adjusted by dividing by the sum of weights of edges to control for false positives. The *p* value of the test is thus given by the following:

$$p=1-\sum_{w_{ij}=0}^{w_{ij}^{c}}g.$$ (8)

In order to determine whether a significant tie *w*_*ij*_ exists between nodes *i* and *j*, we simply count the number of times that the temporal link between them was found to be significant according to the threshold value *α*. If the count of significant links between *i* and *j* falls above half of the *τ* time intervals, that is, *count* > $\frac{\tau }{2}$, the link *w*_*ij*_ is retained in the backbone network. Note that the final backbone network is a binary network, meaning that the weight of links is 1 if the link between two nodes is determined to be significant, and 0 otherwise. However, a weighted network of significant ties can be easily created through various error measures such as averaging the difference between the weights of the temporal links $w_{ij}^{t}$ and the *c*th percentile weight $w_{ij}^{c}$ of the distribution.

**Figure 2.**
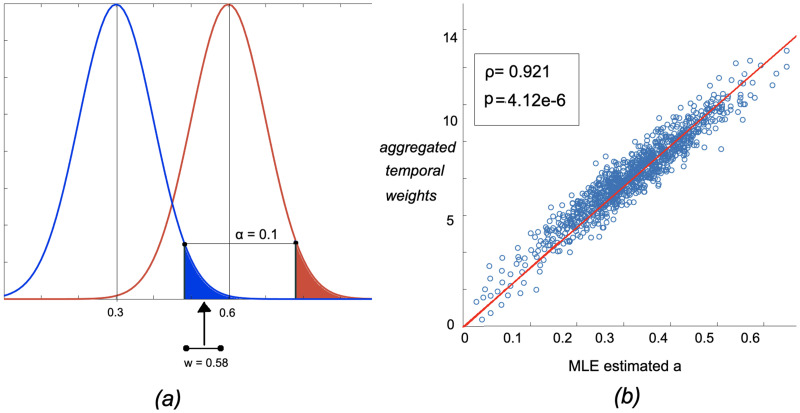
Controlling the effect of local strenghts of node and edge weight on admissibility of the link to the backbone network. (A) An example of the effect of estimated *a* (distribution mean parameter) on admissibility of an empirical temporal link *w*, where the threshold *α* is 0.1 (90th percentile). A link with a low weight can be admitted to the backbone network as long as its estimated mean is sufficiently low (the blue distribution), therefore controlling for the effect of high intrinsic distribution weights on acceptance to the backbone network. (B) Correlation between estimated latent distribution mean variable for each node *i* (*a*_*i*_) and the aggregated dFC weights corresponding to the node over *τ* time steps for the left hippocampus. The weighted degree-estimated *a* pair values are averaged across all subjects within the study dataset.

An important property of the proposed null model is that the tie between two nodes at time *t* can be significant even if the weight of temporal link $w_{i,j}^{t}$ is small, with the condition that their individual latent variables *a*, *b*, and in turn the mean and standard deviation of their temporal distribution, are sufficiently low. On the contrary, ties with large weights might not be deemed significant by WBN if their estimated *a*, *b* are large. This property is illustrated in Figure 2, where large estimated *μ* = *a*_*i*_.*a*_*j*_ shifts the *c*th percentile threshold to the right side of the distribution such that it becomes increasingly difficult for temporal links to meet the threshold.

**Figure 3.**
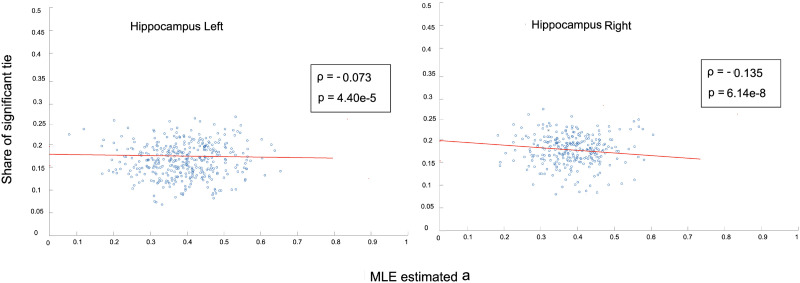
Correlation between share of significant ties connected to each node and the MLE-estimated latent variable *a* for right and left hippocamous regions.

Moreover, strong correlation exists between the MLE-based estimated values of distribution means (*a*_*i*_.*a*_*j*_) and the degree of the nodes, calculated as the sum of weights of the edges over *τ* time intervals. An example of such correlation is shown in Figure 2 for left hippocampus (283 voxels; further empirical results are provided in the Supporting Information), where the aggregated node degree-estimated latent variable *a* were averaged across all subjects of the study data. Also, as Figure 3 shows, there exists a weak negative correlation between the share of significant ties that is connected to each node *i*, and the MLE-estimated variables *a*_*i*_ corresponding to it. The share of significant ties is calculated as the number of ties connected to node *i* that are admitted to the final backbone network divided by the total edges connected to it (*N* − 1). These results establish the property that, based on the WBN model, the admissibility of an edge *w*_*i*,*j*_ to the irreducible network is not attributed merely to its degree, therefore controlling for the effect of local strengths of nodes.

In the next section, we assess and compare the backbone networks detected based on WBN with autoregressive randomization (ARR) as well as phase randomization (PR). ARR and PR are different from ST filter and WBN in the sense that they are applied to the fMRI time series of each temporal window before drawing the connectivity maps of the brain regions, and they are used to explain the fluctuation in generated FC links. ARR assumes that the fMRI data at time *t* is a linear combination of the fMRI data from the previous *p* time points:

$$x_{t}=\sum_{l=1}^{p}A_{l}x_{t-l}+\epsilon_{t},$$ (9)

where *p* ≥ 1, *x*_*t*_ is the *N* × 1 vector of fMRI data at time *t*, and *ϵ* corresponds to zero-mean Gaussian noise, and *A*_*l*_ is an *N* × *N* matrix of model parameters that contains the linear dependencies between each time *t* and its previous time point. ARR first estimates the model parameters for each time point (*A*_1_, …, *A*_*p*_) from the fMRI data. Each null fMRI time series is generated by randomly selecting *p* successive time points from the original data, and then applying the ARR model to generate *Tp* new time points until time series of length *T* are generated. Naturally, significant deviation of the original data from ARR null data means the null hypothesis is rejected.

The PR procedure initiates the null time series generation by performing discrete Fourier transform (DFT) of each time course and adds a uniformly distributed random phase for each frequency, and then same random phase is added across all variables. Finally, an inverse DFT is performed to obtain the null time series. PR generates data with linear, weak-sense stationarity (WSS), and Gaussian properties whose auto-covariance sequence *R*_0_, …, *R*_*T*−1_ is similar to those of the original time series. A rejection of the null hypothesis based on the two mentioned null models could be due to the fMRI time series not possessing either one of the three properties of the null data or a combination of them. The experimental results for WBN as well as the mentioned baseline approaches are provided in more detail in the next section.

## EXPERIMENTAL RESULTS

In order to assess the proposed methodological framework, we apply it to a resting-state fMRI dataset of 300 subjects from the Autism Brain Imaging Data Exchange (ABIDE) database, including 150 subjects diagnosed with autism spectrum disorder (ASD; Di Martino et al., 2014). This dataset was selected from the C-PAC preprocessing pipeline and was slice time and motion corrected, and the voxel intensity was normalized using global signal regression. The automated anatomical labeling (AAL) atlas was then used for parcellation of regions of interest (Tzourio-Mazoyer et al., 2002). Then, the temporal links between each pair of nodes were extracted based on the Pearson correlation between their BOLD activation time series within each temporal window *t*, and were then rescaled based on min-max feature scaling to have continuous values within the range [0, 1]. The implementation code for the methodology in this work is available at https://github.com/ThisIsNima/Weighted-Backbone-Network (Asadi, Olson, & Obradovic, 2021).

After extracting the backbone networks, we probed several aspects and measures of them that will be discussed in this section. In particular, we provide a closer assessment of backbone networks on four brain regions, namely the left and right hippocampus and the left and right amygdalas. We also provide part of the experimental results for the cerebellar regions in the main manuscript and the rest in the Supporting Information. The reason for choosing these regions is the extensive focus of prior literature related to diagnosis and pattern discovery in functional connectivity among ASD patients on them and the fact that several types of abnormality have been discovered related to these regions among this group of patients (Cooper et al., 2017; Guo et al., 2016; Ramos, Balardin, Sato, & Fujita, 2019; Rausch et al., 2016; Shen et al., 2016).

As the first step of our analysis, we examined the normality of the distribution of temporal links between each pair of nodes *i*, *j* across the experiment time *τ*. For this purpose, we used the Kolmogorov-Smirnov test on temporal ties between each pair of nodes for four different window sizes Δ ∈ {5, 10, 15, 20}. Table 1 demonstrates the average *p* values of the normality tests for the distribution of temporal ties between every pair of voxels across 300 subjects for four separate regions. These results demonstrate that the *p* values are below the 0.005 common threshold for rejecting the null hypothesis. Furthermore, the *p* value tends to increase as the size of temporal windows decreases, which can be attributed to the increase in the total number of temporal windows *τ*. Beyond the theoretical basis of the central limit theorem, these results further highlight that the assumption of normality for the distribution of temporal edges in our resting-state fMRI data is reasonable.

**Table 1.** *P* values of Kolmogorov-Smirnov test for normality of the distribution of temporal links. The *p* values presented in the table are averaged across all links ((*N*(*N* − 1))/2 edges for *N* nodes) of the network.

Brain region	Δ = 5	Δ = 10	Δ = 15	Δ = 20
L hippocampus	2.1004e−11	2.4801e−9	1.1422e−22	2.5488e−32
R hippocampus	8.1161e−13	2.5102e−10	1.1084e−9	1.1100e−9
L amygdala	6.3835e−14	1.3560e−9	1.1609e−9	1.1102e−9
R amygdala	3.3875e−10	5.0045e−7	1.4108e−7	3.0545e−7

**Figure 4.**
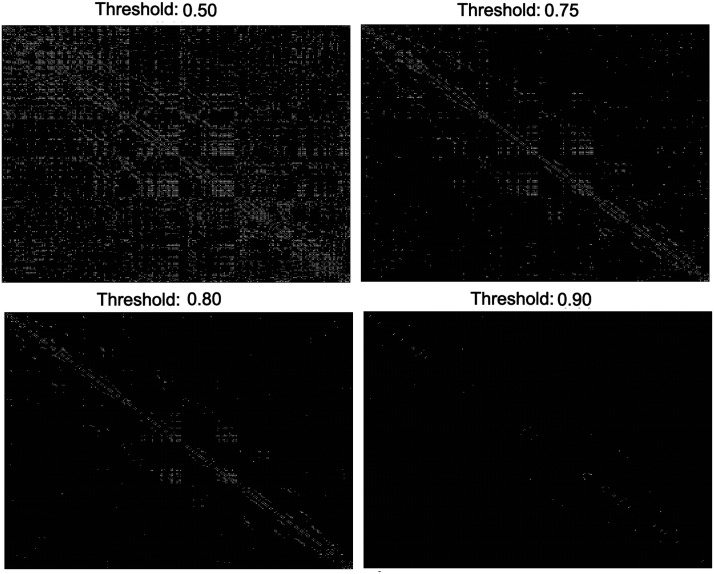
Derived backbone networks of the right hippocampus from one control subject given four threshold values.

The backbone networks of the right hippocampus (region 38 per the AAL atlas) for one control subject based on four different threhsolds (*c* = 1 − *α*) are provided as heatmaps in Figure 4, where each cell represents a voxel, and white cells represent the significant ties. Note that self links are removed from these networks, thus the value of the diagonals of the heatmaps are set to 0. For this analysis, time courses were segmented into 20 temporal windows through the sliding-window approach, with an overlap of 5 time points between consecutive windows. (This is the default setup for the other parts of the experiments. Otherwise, we denote the temporal window size setup.) The visualizations in Figure 4 indicate that the number of admitted links decreases by increasing the threshold *c*. Moreover, the links between voxels in the vicinity of the diagonal line tend to endure the increase in threshold *c*, which highlights the strength of links between spatially close voxels.

The relation between the threshold *c* and the number of significant ties is further inspected in Figure 8-c in the Supporting Information, where the threshold increases from 0.5 to 1 with a fixed step resolution of 10^−2^. The number of significant ties for the network within each time window *t* = 1, …, *τ* is also provided in Figures 8-a and 8-b, where the red bars show the number of edges admitted to the final backbone network. As noted earlier, only the ties that meet the significance threshold in over 50% of the time steps *τ* qualify to be included in the final backbone network (red bar), thus the number of admitted links is usually smaller than the significant ties within various temporal windows. However, as Figure 8 in the Supporting Information demonstrates, the number of significant ties does not demonstrate a large variation across different temporal windows.

**Figure 5.**
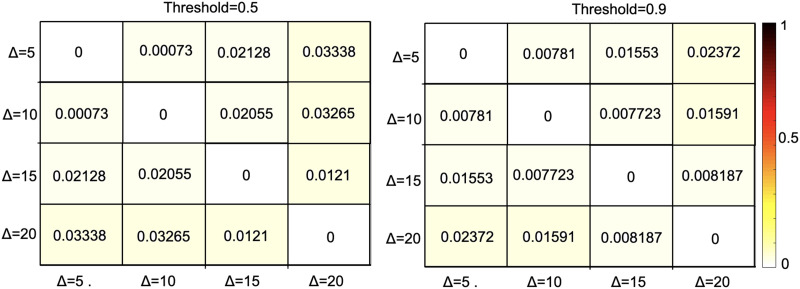
A comparison based on different window sizes using mean percentage error (MPE) of the voxel-wise difference between the backbone networks of dFC averaged across 300 subjects.

For the next step of the analysis, we examined and compared the backbone networks of the two cohorts (control and ASD) within our experimental dataset with similar temporal segmentation as the previous step. For this analysis, the value of *α* was set to 0.2, that is, *c*th percentile = 0.80. Figures 1 and 2 in the Supporting Information present the networks of significant ties extracted from the dynamic connectivity of the left and right hippocampus from four subjects, including two subjects diagnosed with ASD, and Figures 3 and 4 in the Supporting Information show the extracted significant ties from the left and right amygdalas for eight subjects, four of whom were diagnosed with ASD. Moreover, in order to provide a more comprehensive perspective of the irreducible networks of the mentioned regions, the averaged backbone networks of the two cohorts (control and ASD) across the entire dataset are presented in Figure 5 and 6 in the Supporting Information.

As mentioned in the Introduction, several null models have been applied to fMRI connectivity data in the past based on null time series generation. Among these models, autoregressive randomization (ARR) and phase randomization (PR) have been two of the most widely focused approaches. Therefore, we compare the backbone networks based on those two methods with WBN (Handwerker et al., 2012; Liegeois, Laumann, Snyder, Zhou, & Yeo, 2017; Liegeois, Yeo, & Van De Ville, 2021). A comparison of the backbone network extracted through the WBN approach with ARR and PR null models is provided in Figure 7 in the Supporting Information, where the averaged backbone networks of the two cohorts for the right hippocampus are provided based on each null model. We can see that compared with the backbone networks in Figure 5, despite the fact that the backbone networks based on ARR and PR demonstrate a higher density of weights around the diagonals, their averaged values are dispersed across the regions with lower average values. This means that ARR and PR demonstrate a lower consistency of null hypothesis rejection across the subjects in this study compared with WBN. Moreover, WBN demonstrates higher accuracy in detection of randomly injected edges, which will be discussed in the next sections. An explanation for these results can be the fact that in WBN the global and spatial information of the network are considered in latent parameters of each node because of their dependency on the parameters of every other node in the network, which can result in a more stable null model. Another reason can be the fact that the resting-state time courses of different regions can demonstrate variations in statistical properties (Guan et al., 2020; Gultepe & He, 2013). Moreover, stationary linear Gaussian (SLG) models might lack the ability to explain more complex aspects of fMRI dynamics. These issue can particularly intensify in case studies with higher spatial resolution such as voxel-level analysis.

Furthermore, we assessed the effect of the length of temporal windows on the extracted significant ties. For this purpose, we measured the difference between backbone networks of dFC based on four different window sizes: Δ ∈ {5, 10, 15, 20}, where the overlap between consecutive windows was 2 time points for the smallest window (Δ = 5), and 5 time points for the other three window sizes. As the measurement of dissimilarity, we used the mean percentage error (MPE) of the voxel-wise difference (between the values of corresponding matrix cells) between the backbone networks averaged across 300 subjects. The results of this analysis are provided in Figure 5 for two threshold values of 0.5 and 0.9. As this analysis demonstrates, the dissimilarity between the extracted backbone networks calculated as MPE is negligibly small for both temporal resolutions, which indicates the consistency of the backbone network against variations of the temporal window size.

In order to evaluate the effect of the choice of temporal segmentation criteria on the backbone networks, we compare the networks based on sliding-window criteria as well as a change point detection (DCR) approach for single-subject data (Cribben, Wager, & Lindquist, 2013). The DCR approach proposed by Cribben et al. detects the data partitions with the smallest combined Bayesian information criterion (BIC) score to obtain the candidate change points (Cribben et al., 2013). For this analysis, we assigned the value of Δ (the minimum possible number of time points between adjacent change points) to be 10 time points. By comparing Figures 5 (based on sliding window) and 9 (based on DCR) in the Supporting Information, we can note an overall similar backbone structure between the networks based on the two segmentation approaches.

**Figure 6.**
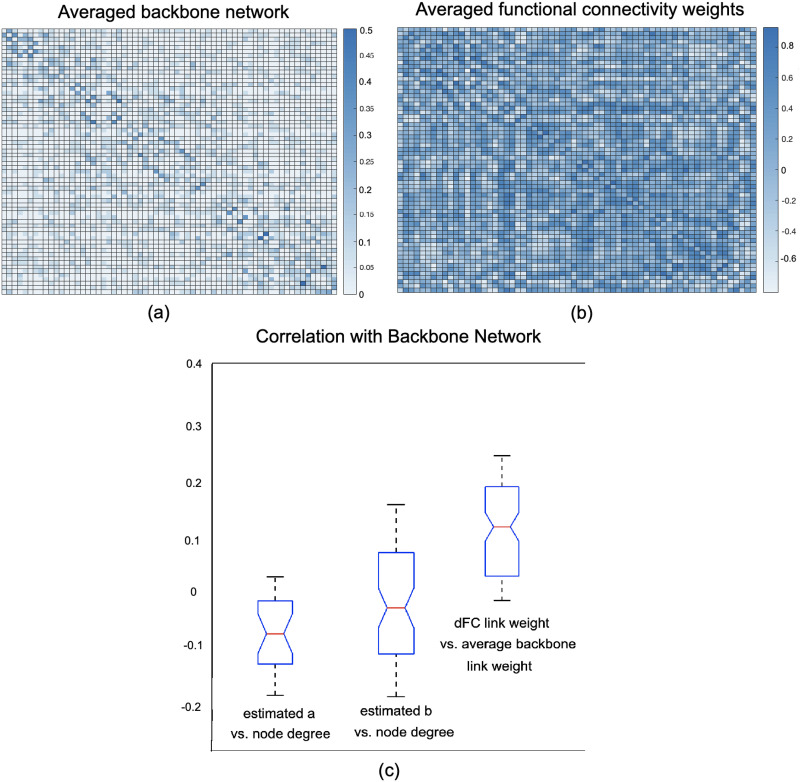
Comparison between the average backbone network and average dynamic functional connectivity weights. (A) Average backbone network of the right amygdala for 300 subjects. (B) Average dFC network of the same region for the same subjects across *τ* = 20 time intervals. (C) Correlations between node degree of the backbone network and estimated a, b as well as the correlation between the backbone network weight and averaged dFC link weight over *τ* = 20 intervals for 300 subjects.

As the last part of the voxel-level experiments, we examined the correlation between the empirical weight of the links and degree of the nodes in the dynamic functional connectivity network with the backbone link wights and estimated latent variables *a*, *b*. In Figures 6A and 6B, the average backbone network of the right amygdala of 300 subjects as well as their average dFC over *τ* windows are presented. Additionally, the correlations between node degrees of the backbone network, calculated as the sum of the link weights for each node, and their estimated a, b as well as the correlation between the average backbone link weights of 300 subjects and the average weight of their corresponding dFC links over *τ* windows are provided in Figure 6C. Results for additional regions are provided in the Supporting Information. As these results demonstrate, there is a weak correlation between the weight of the dFC links and the average weight of backbone links (note that averaging binary backbone links results in continuous weights). Additionally, there is a relatively small negative correlation between node degree of dynamic functional connectivity and estimated distribution latent variables *a*, *b*. In line with the argument provided in the Methodology section, these empirical results further illustrate that WBN considers global and temporal information of the network beyond the local node degree and the weight of the links in the dFC.

### Full-Brain Analysis

**Figure 7.**
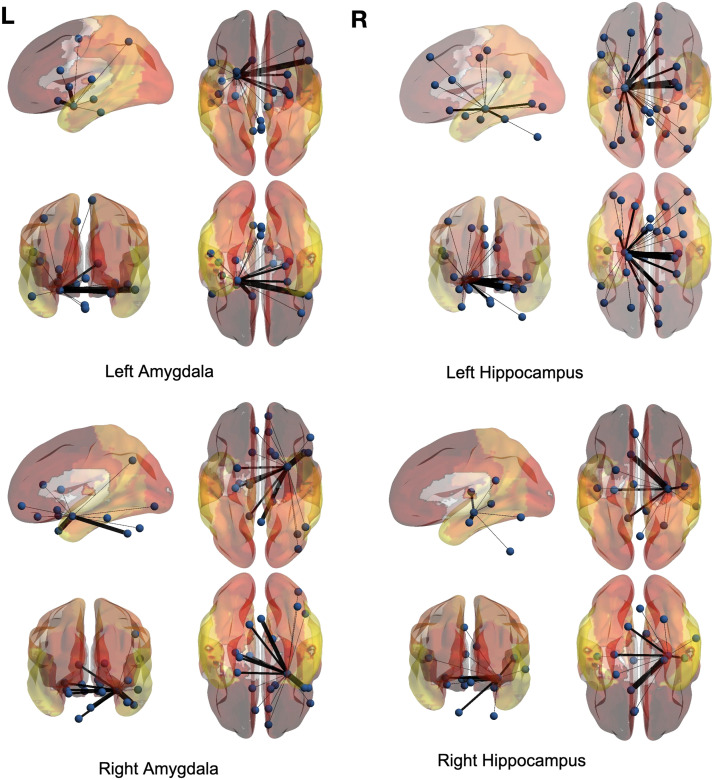
The averaged full-brain backbone networks of 150 control subjects based on four seed regions.

**Figure 8.**
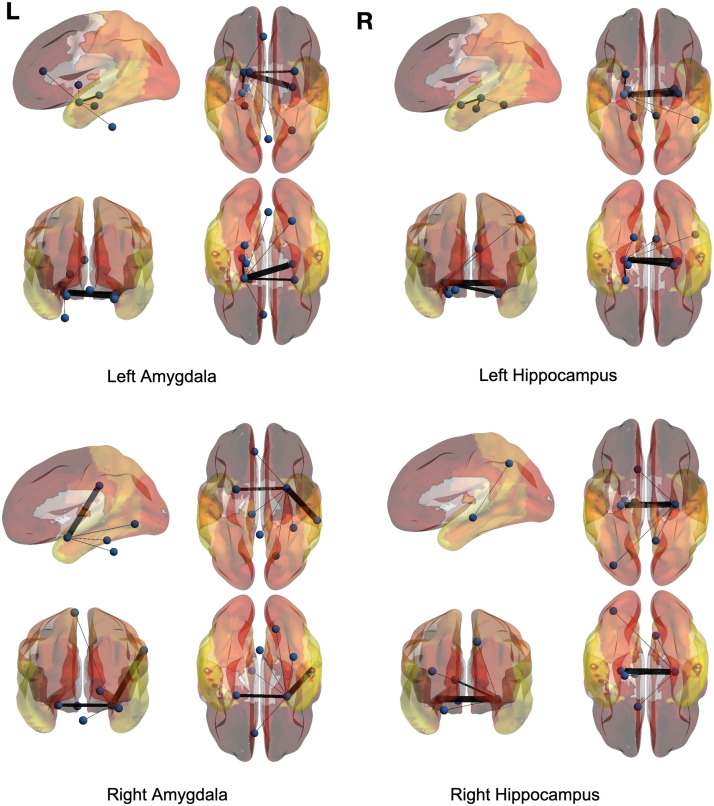
The averaged full-brain backbone networks of 150 ASD subjects based on four seed regions.

**Figure 9.**
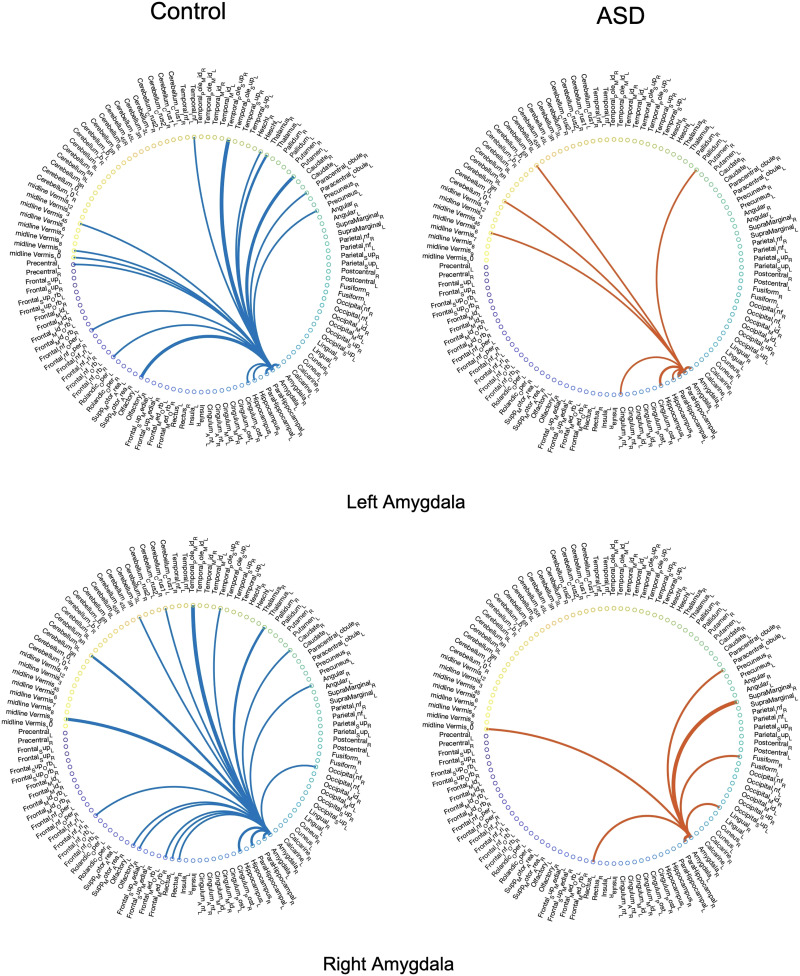
Averaged full-brain backbone networks of 150 ASD and 150 control subjects with the left and right amygdalas as the seed regions.

**Figure 10.**
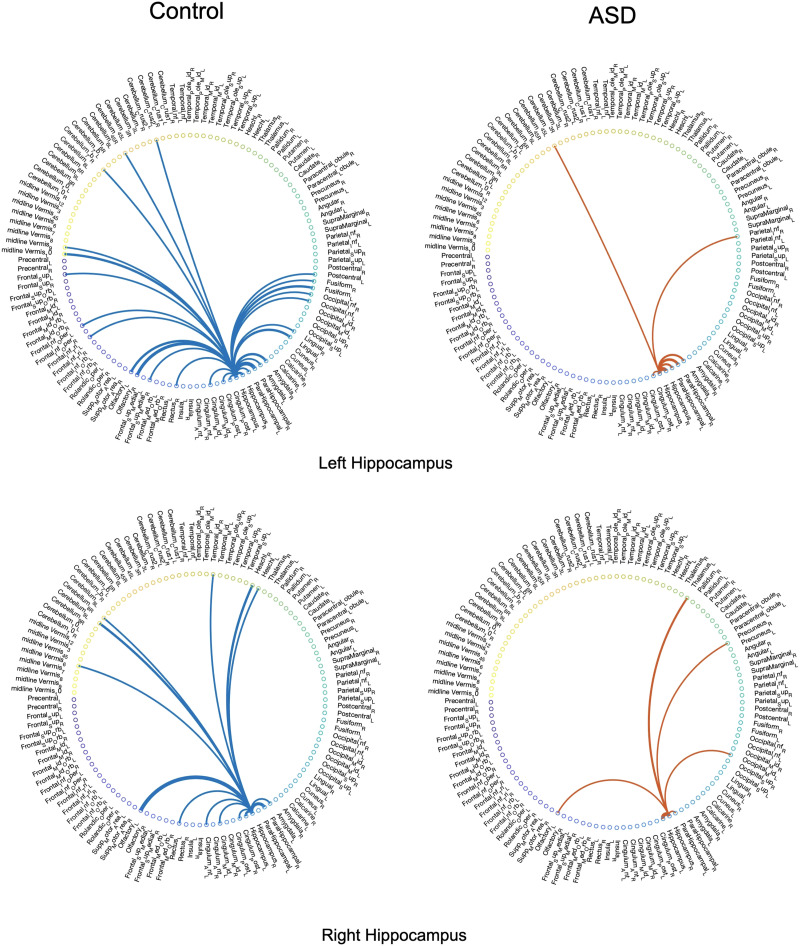
Averaged full-brain backbone networks of 150 ASD and 150 control subjects with the left and right hippocampus as the seed regions.

Just like the voxel-level analysis, a full-brain backbone network of rsFC can be extracted where each node is a region of interest (ROI). For this purpose, the time courses within each region based on the AAL atlas were averaged. The averaged full-brain networks of significant ties for 150 control and 150 ASD subjects are demonstrated in Figures 7, 8, 9, and 10, where the seed regions are the left and right amygdalas and hippocampus, and the average backbone links with weights below 0.05 were filtered out to facilitate easier presentation. In these figures, the link weights (illustrated by thickness in the figure) correspond to the count of their corresponding links appearing in the binary backbone networks across each cohort. We can observe from Figures 9 and 10 that the amygdalas and hippocmapus develop a larger number of significant ties with other regions among the control group compared with the ASD cohort, as the network of the latter cohort is more sparse. The width of links in Figures 7 and 8 also represent the strength of the average correlations. We can therefore also observe a relatively high averaged backbone link between the right and left hippocampus among both the control and the ASD group. However, certain differences can be detected between the two cohorts, including a stronger average backbone tie between the hippocampus and the cerbellums as well as right and left olfactories among control subjects compared with the ASD cohort. Moreover, a higher average backbone tie can be noticed between the left and right amygdalas and the superior and the middle temporal gyrus among control subjects. Further related experimental results are provided in the Supporting Information; these results include the average backbone connectivity with several cerebellar regions being the seed area. These results demonstrate the benefit of the weighted temporal backbone network in revealing the differences in irreducible ties between different regions of interest.

**Figure 11.**
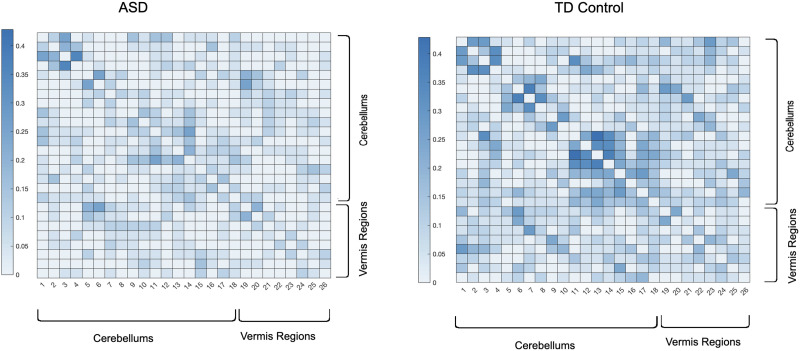
Averaged full-brain backbone networks of cerebro-cerebellar and vermis regions across 150 ASD and 150 control subjects.

Figure 11 depicts the averaged backbone connectivity of the cerebellums (18 regions per AAL) and the vermis (8 regions per AAL) of the two cohorts in this study, which indicates higher connectivity level among the control group compared with the ASD group. These results, along with the experimental results provided in Supporting Information (Figures 5–10), can indicate that the increased cerebro-cerebellar functional connectivity detected in some studies can be driven by a large number of links that fail to be incompatible with the null hypothesis of links being produced at random. In other words, despite the lower connectivity detected in cerebro-cerebellar subnetwork among the control group in terms of number of links or their weights, the number of meaningful and irreducible links in that subnetwork among the healthy cohort tends to be larger compared with the ASD cohort (Khan et al., 2015; Mostofsky et al., 2009; Ramos et al., 2019).

### Detection of Random Links

To compare the performance of the proposed approach with other proposed null models, we considered three different models, namely binary ST filter, ARR, and PR.

**Figure 12.**
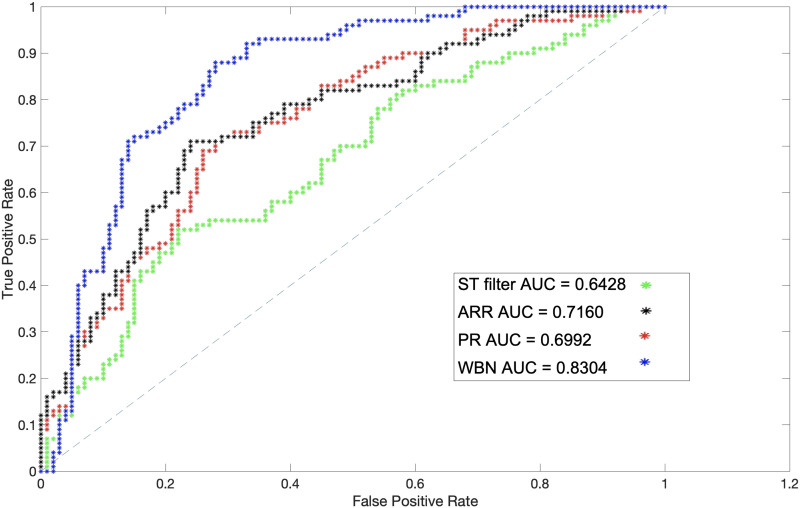
The AUC of detection of injected random weights based on the ST filtering, ARR, PR, and WBN in the left hippocampus.

We created a simulated dataset by injecting random weights to a subset of edges of the real rsFC networks of our dataset. For this purpose, 100 random weights were injected into 100 links of rsFC of the left hippocampus, and the precision of WBN as well as the ST filter approach, proposed by Kobayashi et al., in excluding them from the final network were calculated (Kobayashi et al., 2019). Because the ST filter operates on temporal binary weight networks, in order to evaluate it we converted the rsFC link weights as well as the randomly injected weights into binary links by drawing a temporal link between each pair of nodes whose weight in the original rsFC network was above the entire network’s average. The result of this experiment is provided in Figure 12, where WBN demonstrates an advantage over the ST filter in random link detection precision. A similar experiment with other regions of interest was conducted; these results are provided in the Supporting Information (Figure 20). The evaluation measure for this analysis was calculated by comparing the detection of injected random link weights with the ground truth. Part of the superior performance of WBN can be attributed to the fact that the process of conversion to a binary network for the ST filter setup results in loss of information and precision, which is an inherent disadvantage of backbone network detection approaches that are designed for binary networks.

## DISCUSSION

In this work, we proposed a new approach for detecting the significant ties between nodes on voxel- and ROI-level networks of resting-state dFC. The proposed framework entails two computational steps: First, a maximum likelihood optimization is performed to calculate the latent variables that characterize the optimal Gaussian distribution of the temporal links between each pair of nodes across *τ* time steps. Then, the empirical link weights between each pair of nodes within each temporal window are compared with the *c*th percentile of the Gaussian distribution to detect the significant links that form the backbone network. This process is performed for every pair of nodes in the temporal network of dFC. Aside from providing a systematic filtering framework for weighted temporal networks such as resting-state dFC, this approach has several analytical advantages over other prior filtering approaches that we discuss in this section. We also discuss the limitations of the proposed methodology along with possible suggestions for improvement and future plans.

As mentioned previously, inclusion of a temporal link in the backbone network is determined by testing the hypothesis that the link can be explained by the null model that links are created uniformly at random. This comparison is applied to every link in the dFC individually, that is, between every pair of nodes and within every temporal window. Therefore, temporal properties and variations of the network structure over time are taken into account in backbone network inference. This property is an advantage of the proposed methodology over some of the prior approaches that consider a constant intrinsic activity value for the nodes over time. It also offers the power of determining a cutoff percentage of ties having a larger weight over the *c*th percentile, which was decided to be 50% in this study.

Another advantage of the suggested approach is the fact that it considers the interplay of global and local information of the network in estimating the latent variables *a* and *b*. In other words, the significance of temporal ties cannot be attributed merely to node properties such as degree or centrality measures, because each equation in the system of *N* equations of Equations 4 and 5 takes into account the combination of weights over time for each link for node *i* as well as their combination with other links between *i* and every other node in the network. This property has been discussed in more detail in the Methodology section and evaluated in the Results section.

The refinement of parameters of the distribution through maximum likelihood optimization requires solving the system of *N* equations for *N* nodes (one set of *N* equations for each of the two parameters), which can be solved through several optimization approaches such as gradient-based optimization, search methods, or the Newton method. Solving these equations does not require any hyperparameter tuning, as the only parameters that need to be selected as input are the threshold value *α* and the percentage of times that the weight of the link meets the *c* = 1 − *α* percentile of the distribution, which offer the flexibility for a comprehensive assessment of the temporal ties in the dynamic connectivity network.

Unlike some of the null models suggested in the past for binary networks based on binomial or Poisson distributions, the methodology put forward in this work does not assume a strictly positive weight between interacting nodes. This property provides the flexibility for ties that are generated through various approaches such as correlation measures to be considered in the null model, as negative correlation is a possibility between interacting nodes.

Another advantage of the proposed approach is the fact that the backbone networks are learned for each subject individually. As explained in the Methodology section, the input for WBN is the weighted dynamic connectivity network of a subject, and its output is the network of irreducible ties corresponding to the subject. This property has the benefit of taking into account the individual differences when inferring the backbone network in an isolated fashion.

The suggested methodological framework can be used in studies with various scales and resolution of dFC networks, meaning that instead of voxel-level analysis, dFC networks consisting of regions of various scales as nodes can benefit from this approach as well. Moreover, this approach is independent of a temporal segmentation step, as long as the statistical properties of independence and normality are met.

### Limitations

Despite the mentioned advantages, the proposed approach bears certain limitations that we highlight in this section.

As discussed in the Methodology section, the first step of the suggested framework entails estimation of latent variables *a* and *b*, which rule the propensities to generate a distribution of links with a certain average and standard deviation. However, these variables are estimated and compared across the experiment time *τ*, that is, the length of the fMRI signals. In other words, the mean and standard deviation of the distributions, and in turn the backbone network calculations, can vary depending on the length of the experiment.

The structural characteristics of dFC can be influenced by temporal fluctuations in the data throughout the course of the experiment. In other words, reducible links might not have the same statistical features at any time during the observation, as node properties might not be constant over time. Therefore, more improvements need to be applied to WBN to take such variations into account.

Despite its adaptability with regards to different temporal segmentation approaches such as sliding window or DCR, WBN requires an equal number of temporal windows across the entire region of interest for calculating the latent distribution variables for each node because of the number of optimization equations that it solves.

Another limitation of the suggested approach is the assumption of normality for larger temporal window sizes. As the empirical tests demonstrated, an increase in size of the temporal windows could in principle weaken the normality assumption of the distribution of the temporal links. Despite the evidence of normality for reasonable and common window sizes in the literature, this assumption needs to be further explored for different datasets.

The MLE optimization for estimating the intrinsic variables *a*, *b* plays the largest role in the computational complexity of the methodology presented in this work. The computation time depends on the number of nodes (i.e., spatial resolution) and the number of time intervals that the signal is segmented to. By definition of the approach, the spatial resolution plays a more significant role in the computational complexity (refer to Equations 4 and 5). In this study, a system of *N* equations were solved through the trust-region-dogleg method, whose computation time for regions below 1,000 voxels was 10 min for 8 GB of RAM. However, more efficient approaches can be employed for this purpose.

Alleviating the mentioned limitations requires further methodological explorations and analytical studies on various datasets. As future work, our objective will include assessing the backbone network of resting-state dFC of other cohorts and data from various neurological conditions and studying different group differences. Furthermore, assessing significant temporal structures and graph communities and motifs as well as exploring the effect of different preprocessing pipelines and temporal sample size on the outcome of the proposed approach can be fruitful paths for further experiments in the area of dynamic functional connectivity.

## SUPPORTING INFORMATION

Supporting information for this article is available at https://doi.org/10.1162/netn_a_00209. The implementation code for the methodology in this work is available at https://github.com/ThisIsNima/Weighted-Backbone-Network.

## AUTHOR CONTRIBUTIONS

Nima Asadi: Conceptualization; Data curation; Formal analysis; Investigation; Methodology; Software; Visualization; Writing – original draft; Writing – review & editing. Ingrid R. Olson: Formal analysis; Investigation; Supervision; Validation. Zoran Obradovic: Formal analysis; Project administration; Supervision; Validation.

## FUNDING INFORMATION

Ingrid R. Olson, National Institutes of Health (https://dx.doi.org/10.13039/100000002), Award ID: R01HD099165. Ingrid R. Olson, National Institutes of Health (https://dx.doi.org/10.13039/100000002), Award ID: RO1 MH091113. Ingrid R. Olson, National Institutes of Health (https://dx.doi.org/10.13039/100000002), Award ID: R21 HD098509. Ingrid R. Olson, National Institutes of Health (https://dx.doi.org/10.13039/100000002), Award ID: 2R56MH091113-11.

## TECHNICAL TERMS

Temporal segmentation: The process of slicing the fMRI time courses into consecutive temporal windows within which connectivity matrices are formed based on the correlation between the fMRI time series. A common temporal segmentation is the sliding-window approach that computes a succession of pairwise correlation matrices using the time courses from a given parcellation of brain regions.
Temporal ties: The link between a pair of nodes whose weight might vary across the time of experiment.
Backbone network: The network that is formed by the significant ties between nodes by filtering out randomly generated edges or noise-induced links.
Null model: Null models are formulated as a baseline for comparison with the system to verify whether the system displays properties that would not be expected on a random basis or as a consequence of certain constraints.
Latent model variables: Model variables that are not directly observed or assigned, but are inferred from other measurements from the data or from variables that are observed.
Newton method: An approach for solving a system of nonlinear equations by finding the roots of differentiable functions.
Weak-sense stationarity: A random process whose mean function and its autocovariance function do not fluctuate by variations in time.

## Supporting Information

https://github.com/ThisIsNima/Weighted-Backbone-Network
