# Supplementary material for: The backbone network of dynamic functional connectivity
Source: Netw Neurosci. 2021 Nov 30;5(4):851–73. doi: 10.1162/netn_a_00209 (PMC8746122; doi:10.1162/netn_a_00209)
Supplement: Supplementary file 1 [file netn-05-851-s001.pdf]

Asadi, N., Olson, I. R. & Obradovic, Z. (2021). Supporting information for "The Backbone Network of Dynamic Functional Connectivity." *Network Neuroscience*. Advance publication.
https://doi.org/10.1162/netn_a_00209

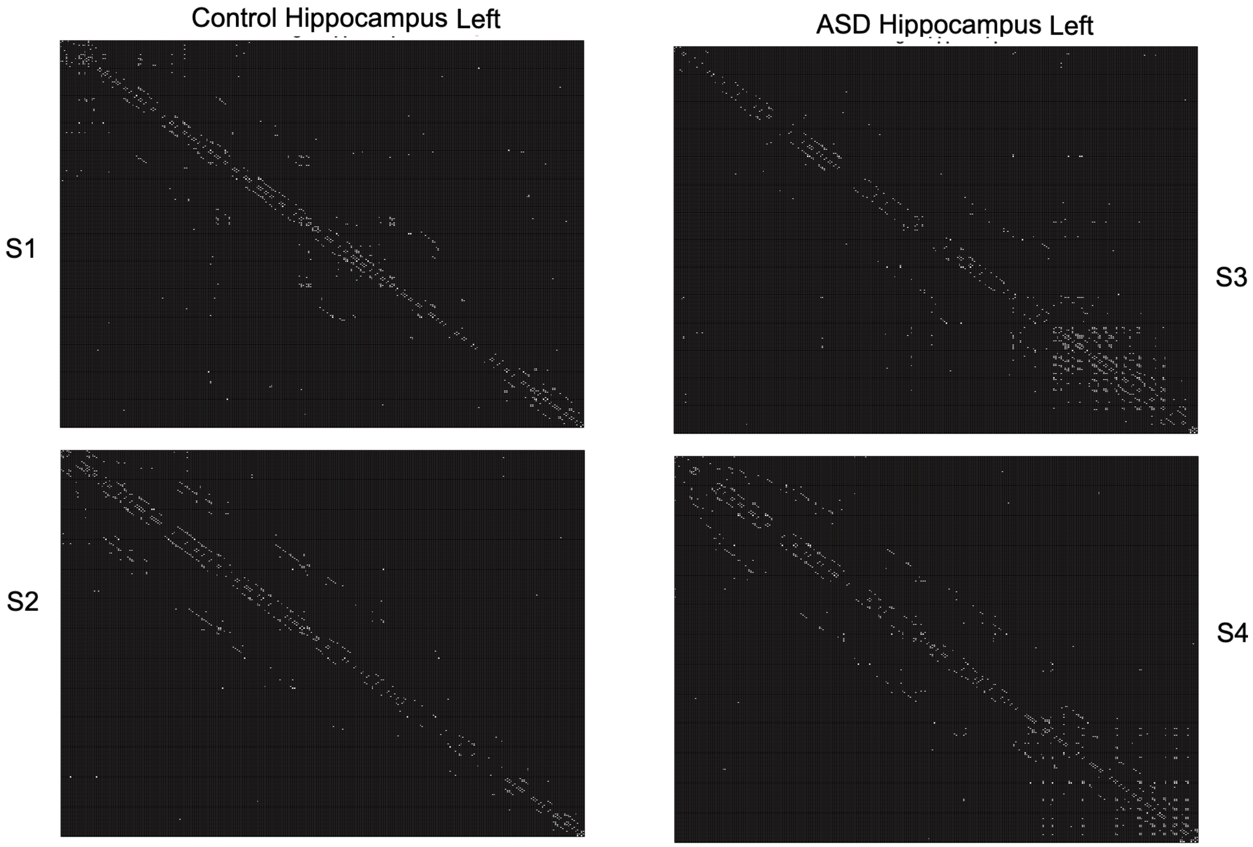

1 **Figure 1.** Extracted backbone network of the left hippocampus from four subjects; two control subjects and two diagnosed with ASD, based on a 0.80
2 threshold.

SUPPLEMENTARY MATERIAL

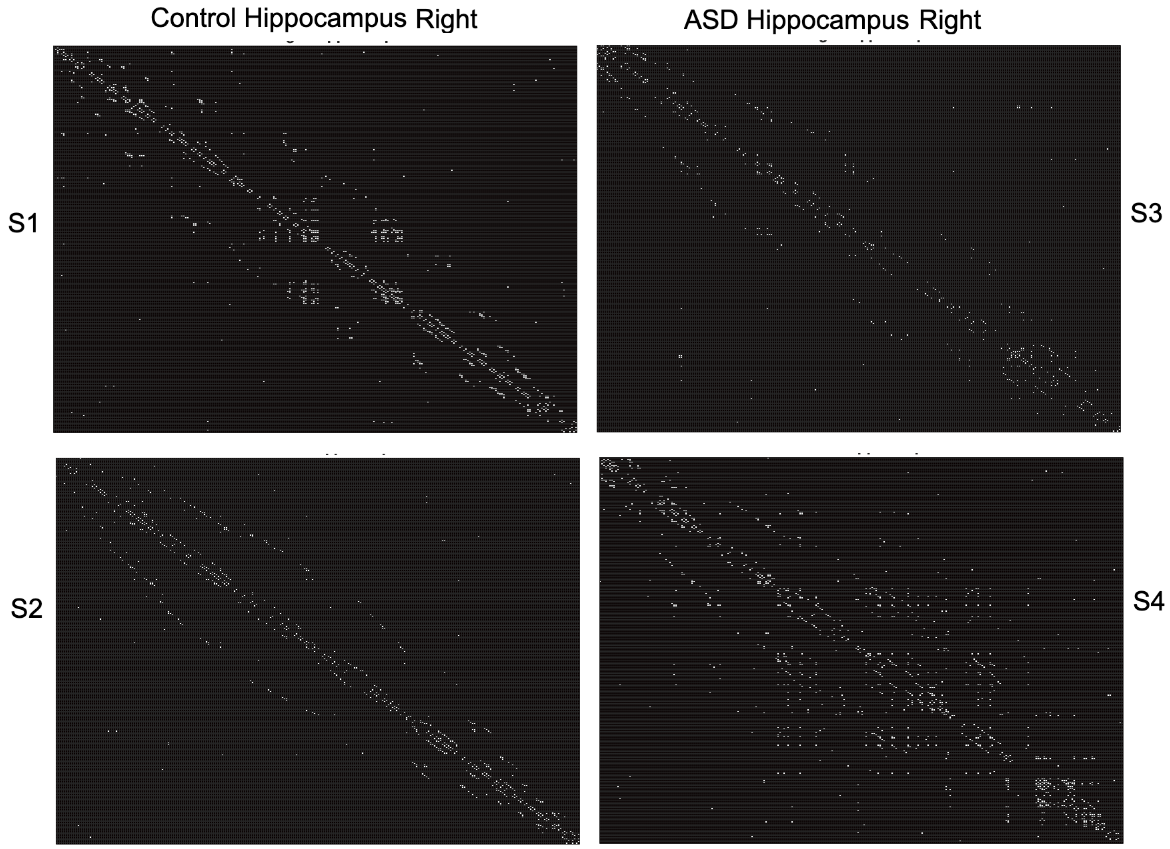

Figure 2. Extracted backbone network of the right hippocampus from four subjects; two control subjects and two diagnosed with ASD, based on a 0.80 threshold.

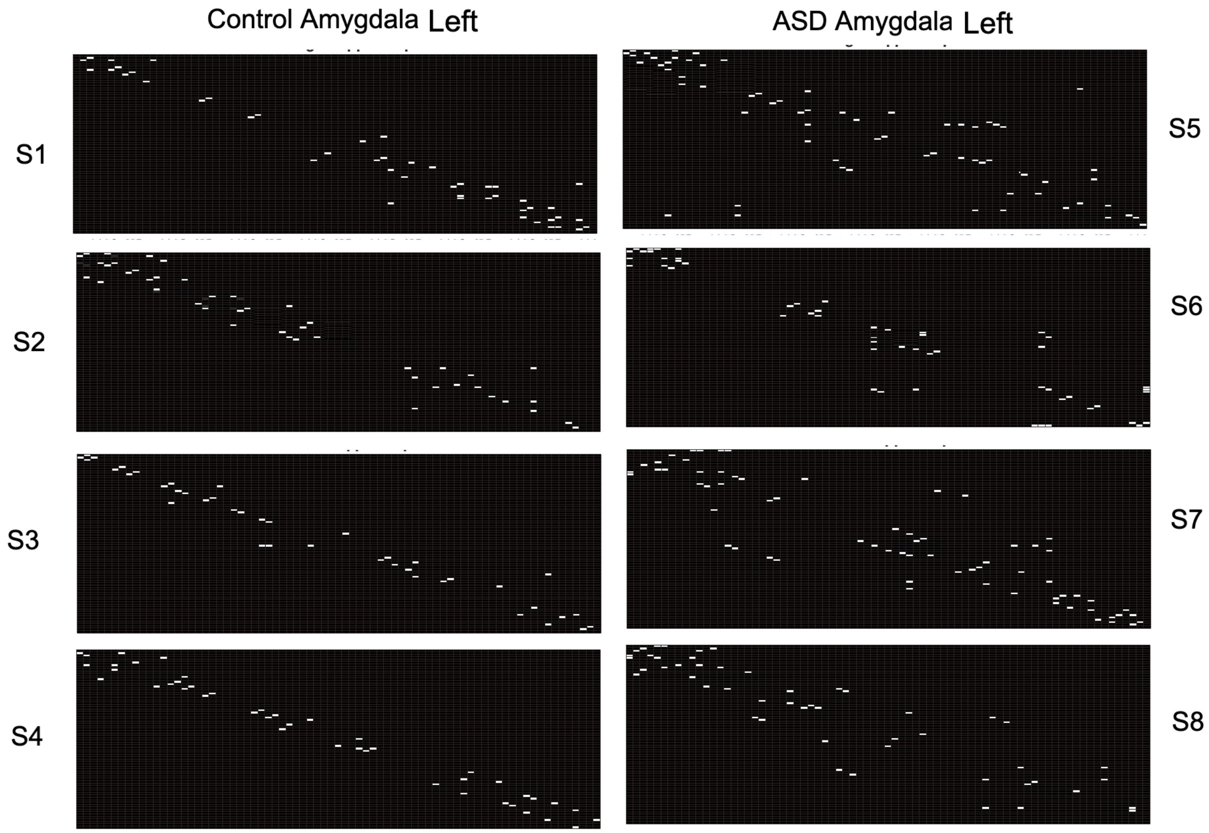

Figure 3. Extracted backbone network of the left Amygdala from eight subjects; four control subjects and four diagnosed with ASD, based on a 0.80 threshold.

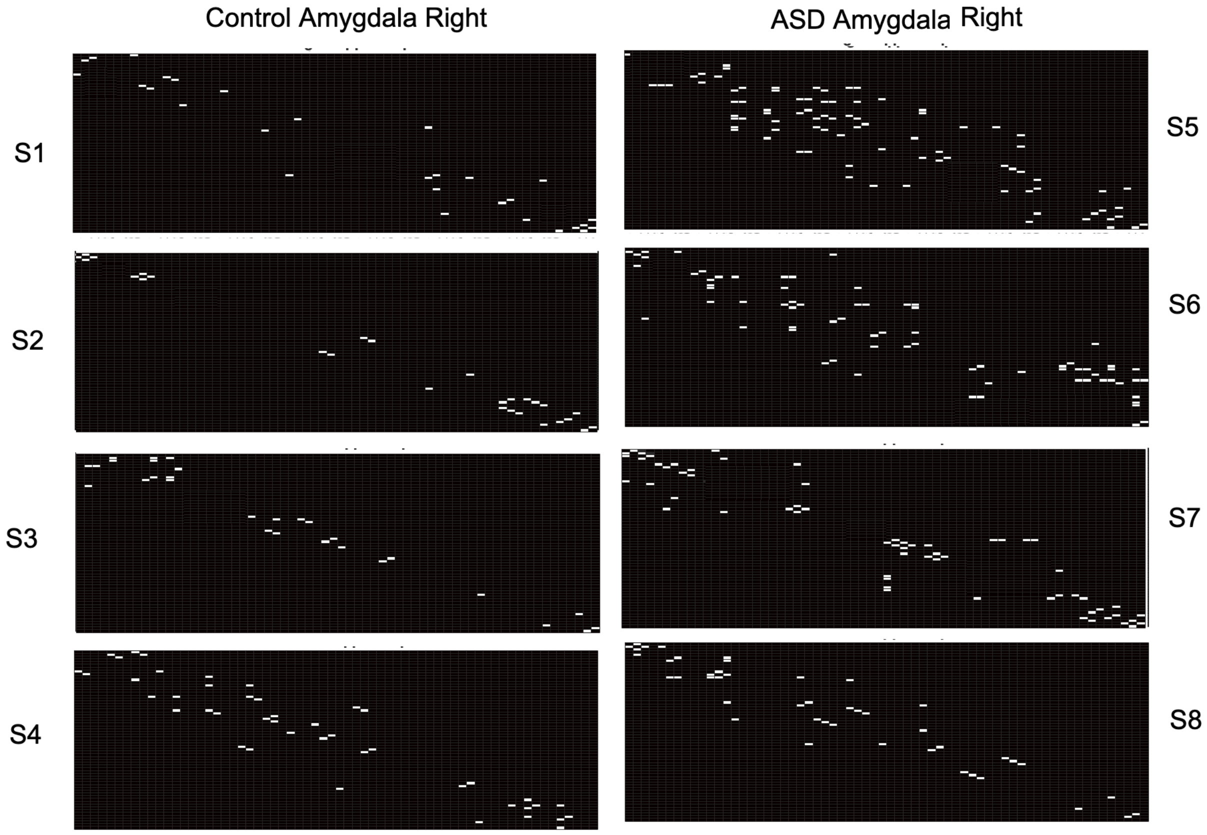

Figure 4. Extracted backbone network of the right Amygdala from eight subjects; four control subjects and four diagnosed with ASD, based on 0.80 threshold.

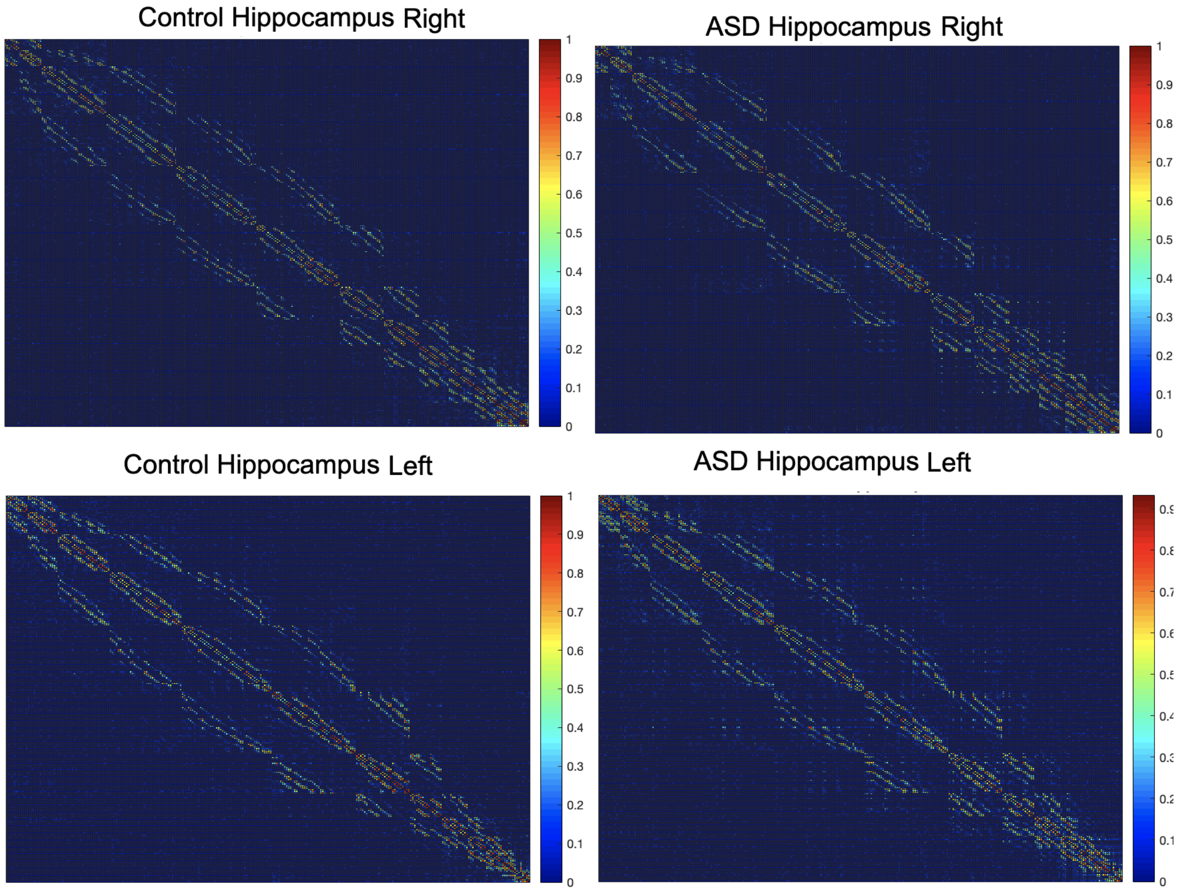

9 **Figure 5.** Average backbone network of the right and left hippocampus from 300 subjects; 150 control subjects and 150 diagnosed with ASD, based on a
10 0.80 threshold.

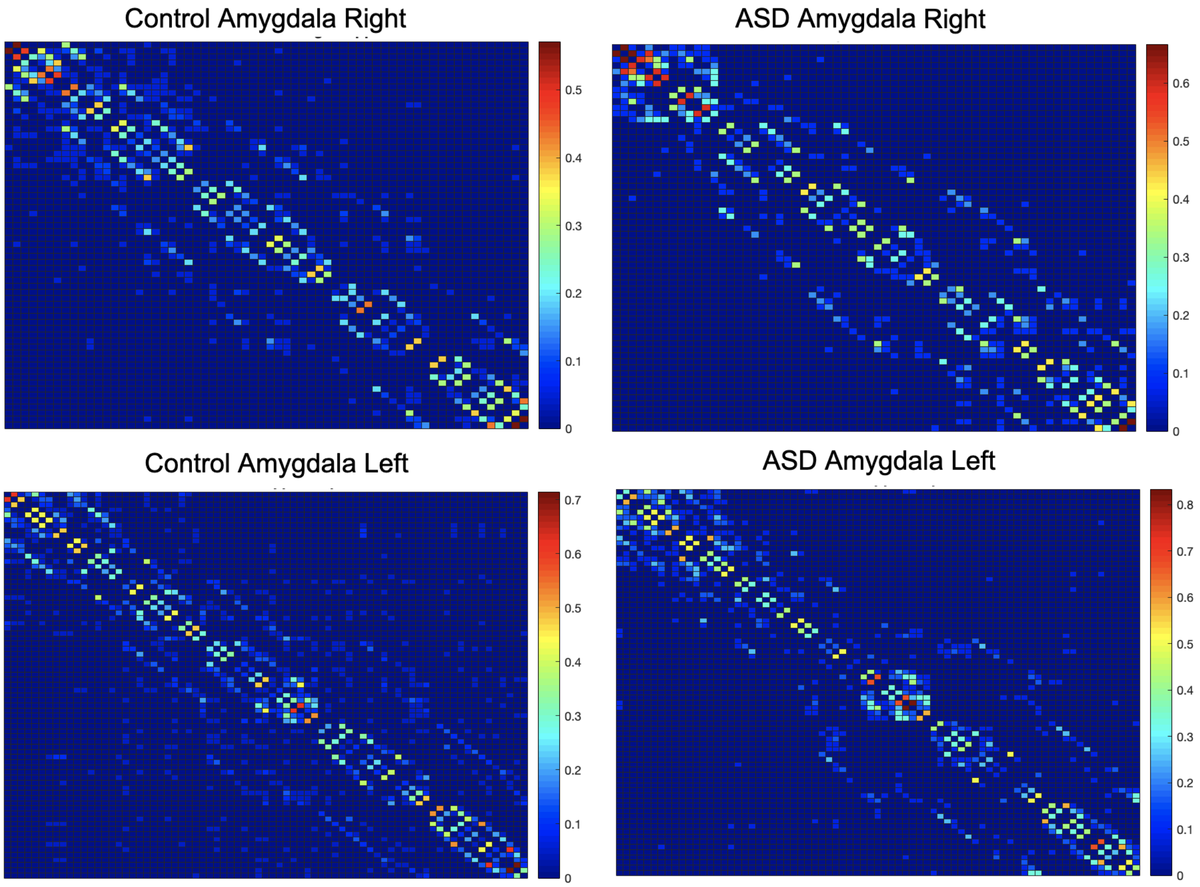

Figure 6. Average backbone network of the right and left Amygdalas from 300 subjects; 150 control subjects and 150 diagnosed with ASD, based on a 0.80 threshold.

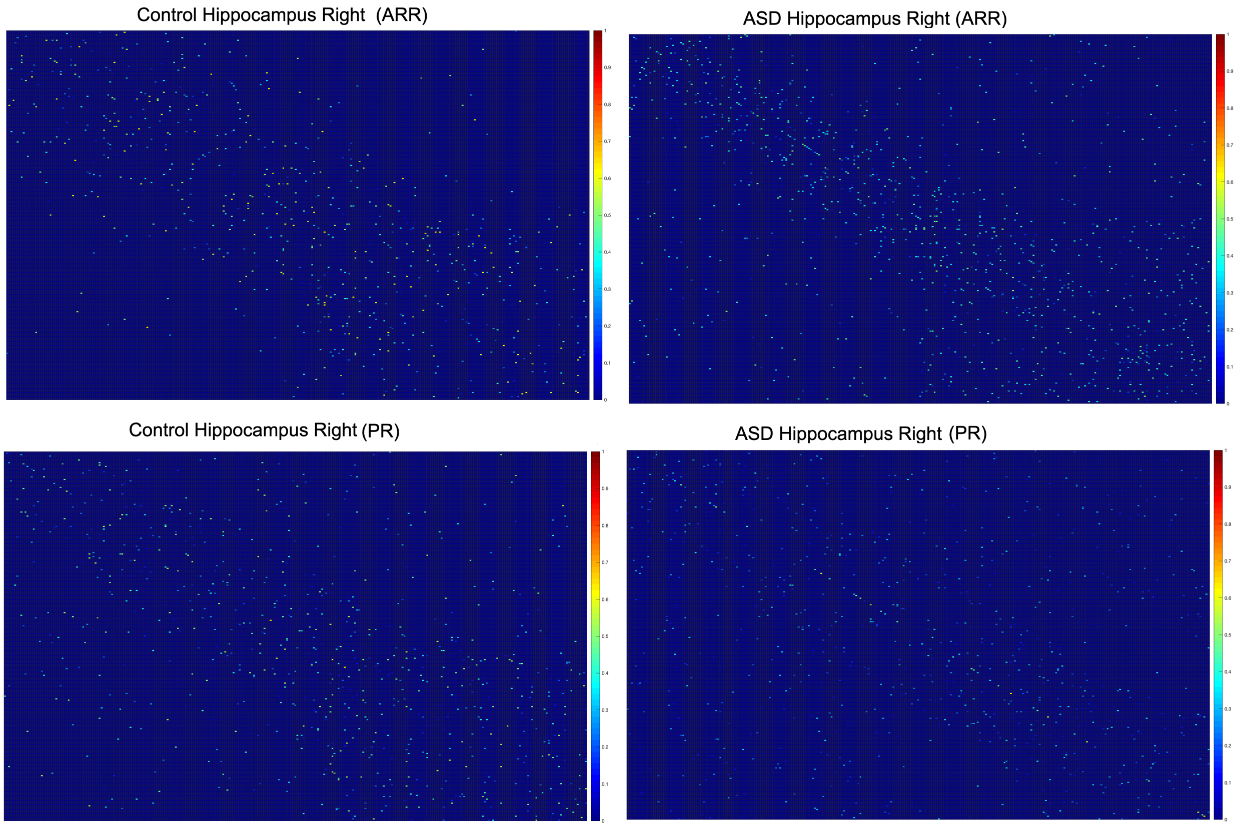

13 **Figure 7.** Average backbone network of the right hippocampus from 300 subjects; 150 control subjects and 150 diagnosed with ASD, based on the ARR
14 and PR null models.

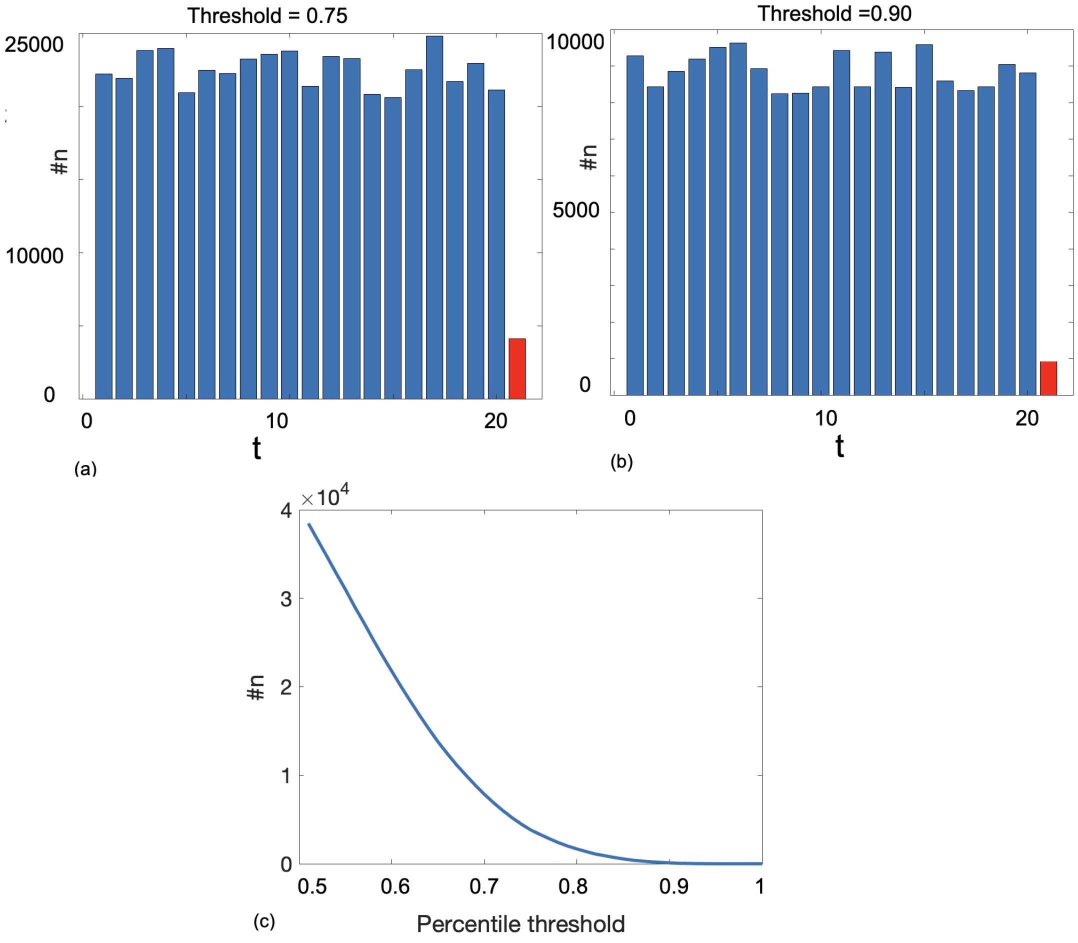

Figure 8. figures a and b: number of significant ties n for the right hippocampus network of one control subject across $t = 1, \ldots, 20$ time steps based on two different threshold values. The red bar corresponds to the number of edges of the temporal network admitted to the backbone network. figure c : the number of admitted edges to the final backbone network based on 50 different threshold values between 0.5 and 1 with fixed resolution.

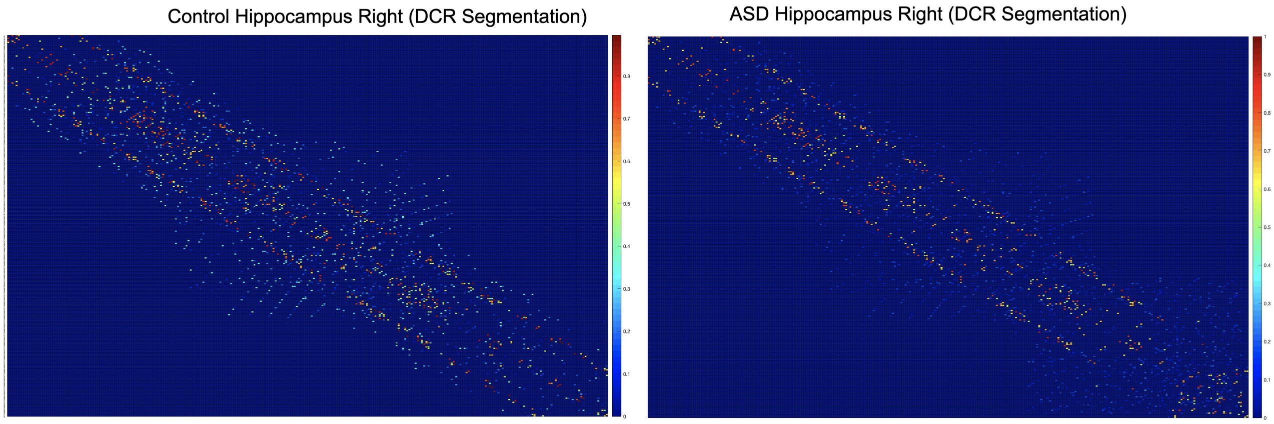

Figure 9. The averaged backbone networks of right hippocampus between the control and ASD cohorts where DCR temporal segmentation was applied.

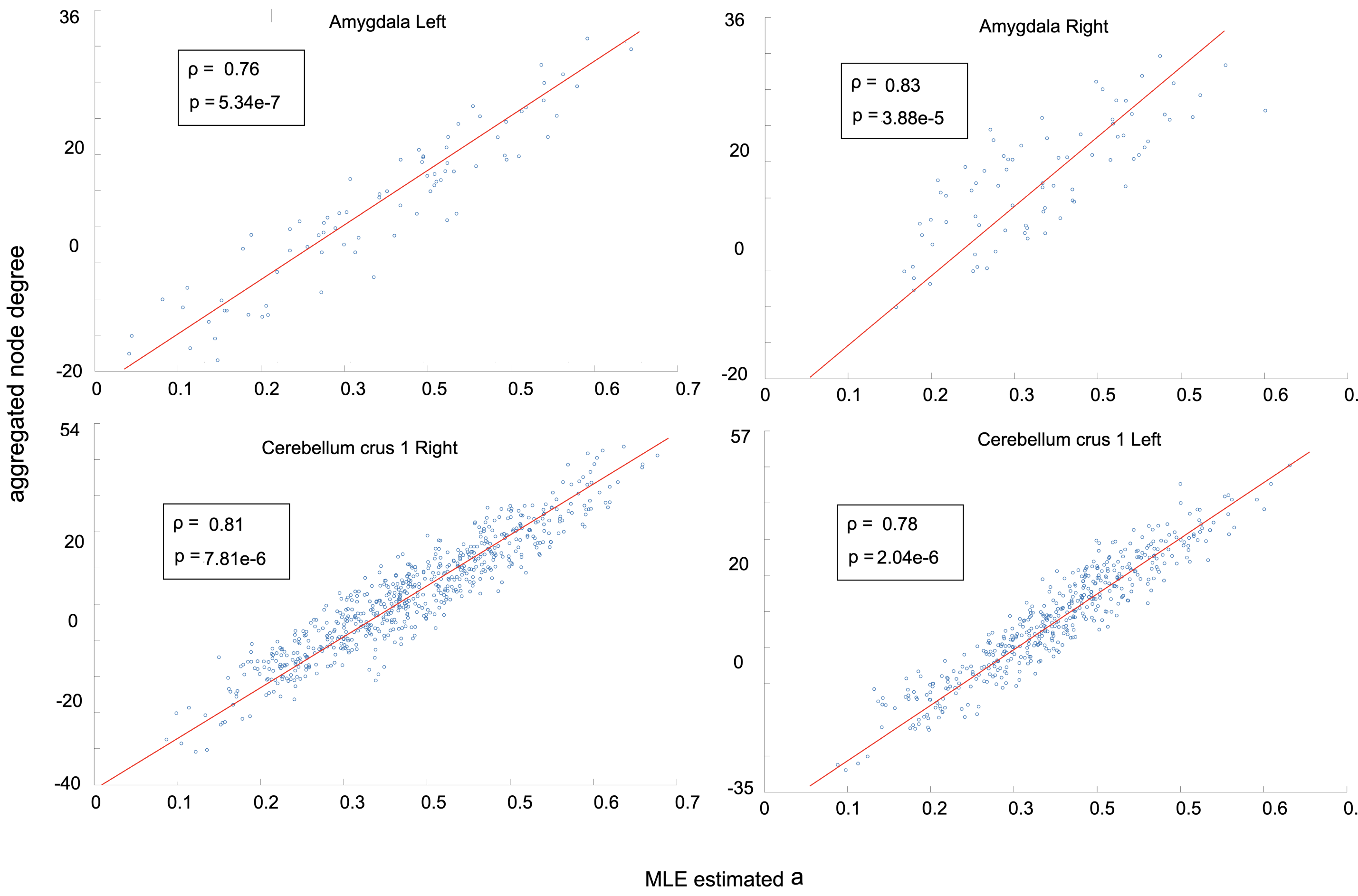

19 **Figure 10.** Correlation between node degree calculated as aggregated weights of all edges connected to each node over time τ and the MLE estimated latent
20 distribution mean variable a

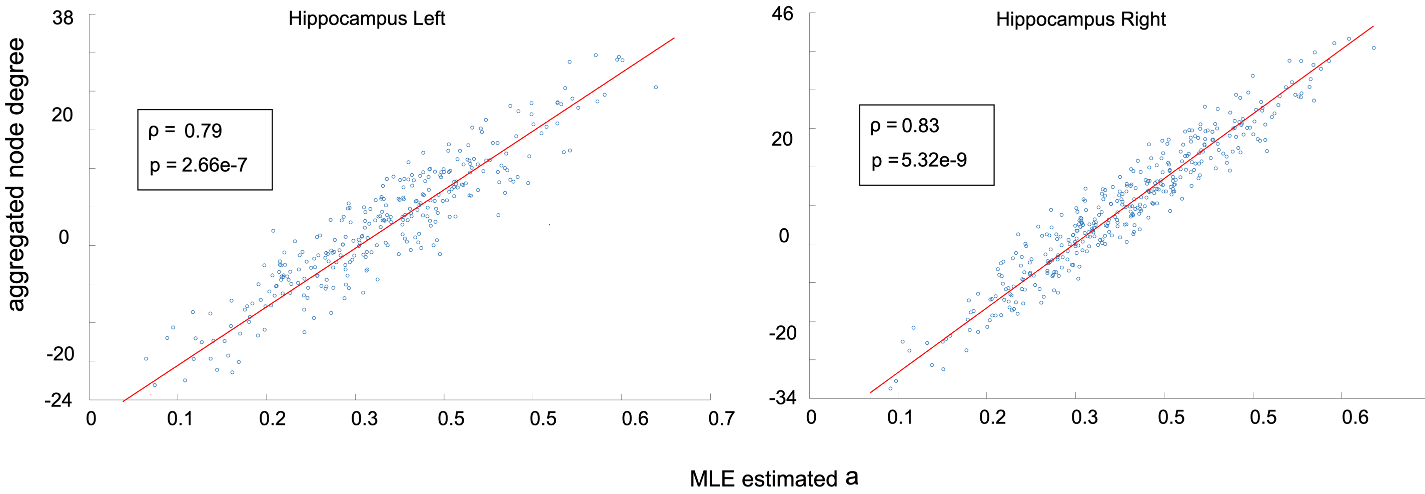

21 **Figure 11.** Correlation between node degree calculated as aggregated weights of all edges connected to each node over time τ and the MLE estimated latent

22 distribution mean variable a

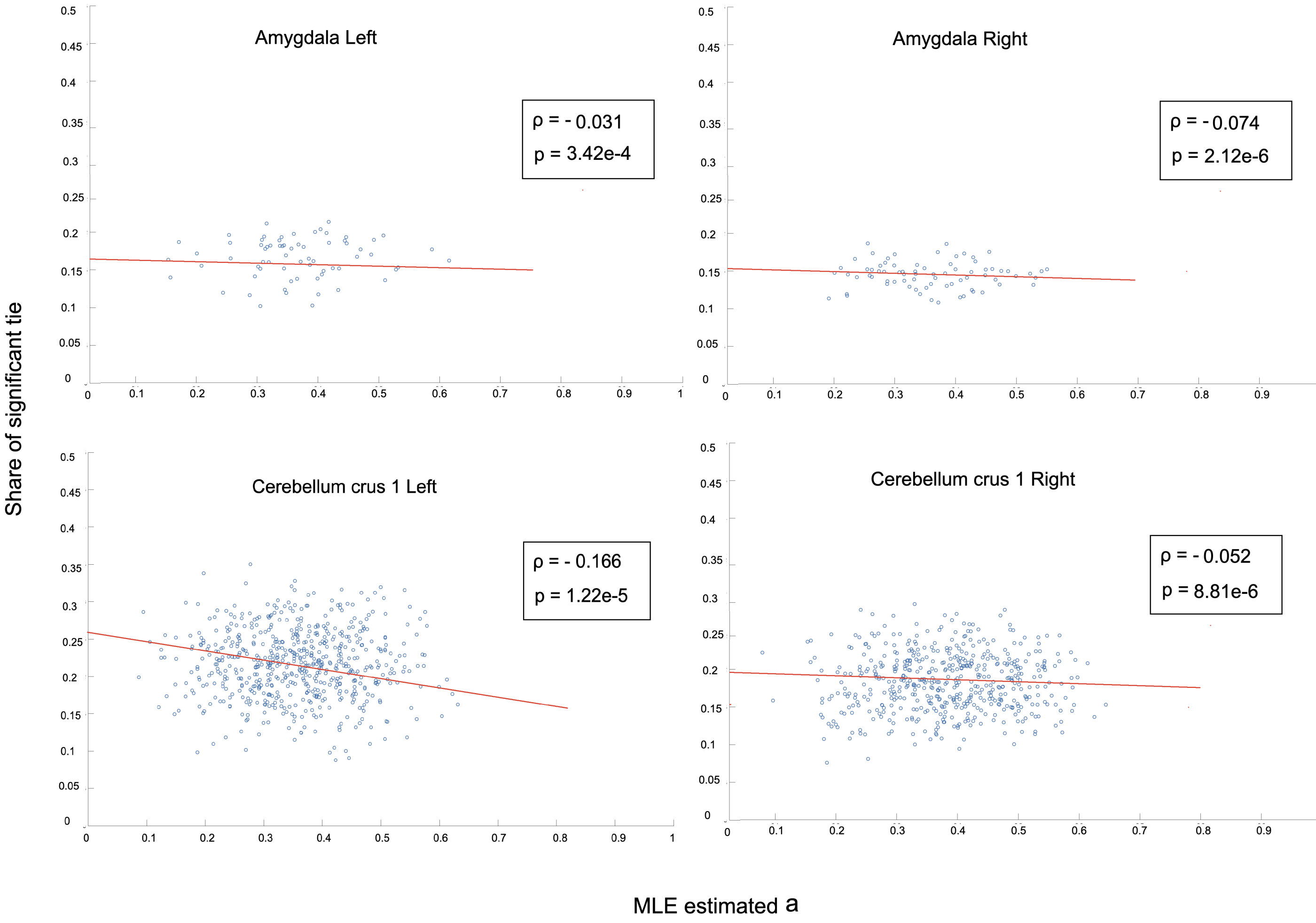

23 **Figure 12.** Correlation between the share of significant ties of each node over time τ and the MLE estimated latent distribution mean variable a

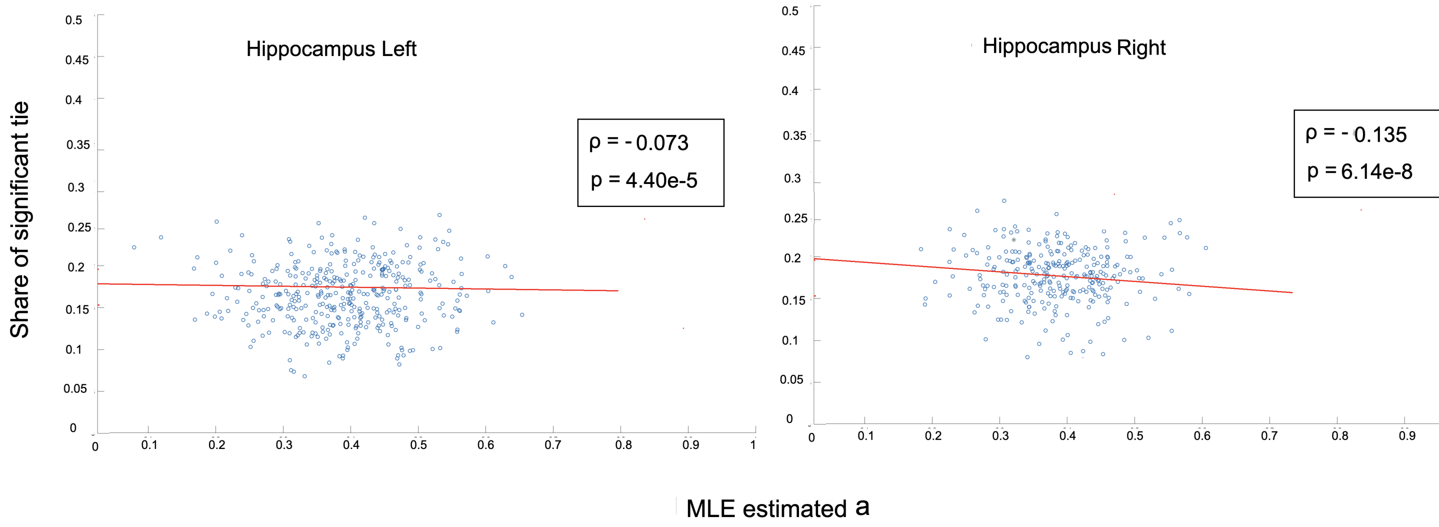

24 **Figure 13.** Correlation between the share of significant ties of each node over time τ and the MLE estimated latent distribution mean variable a

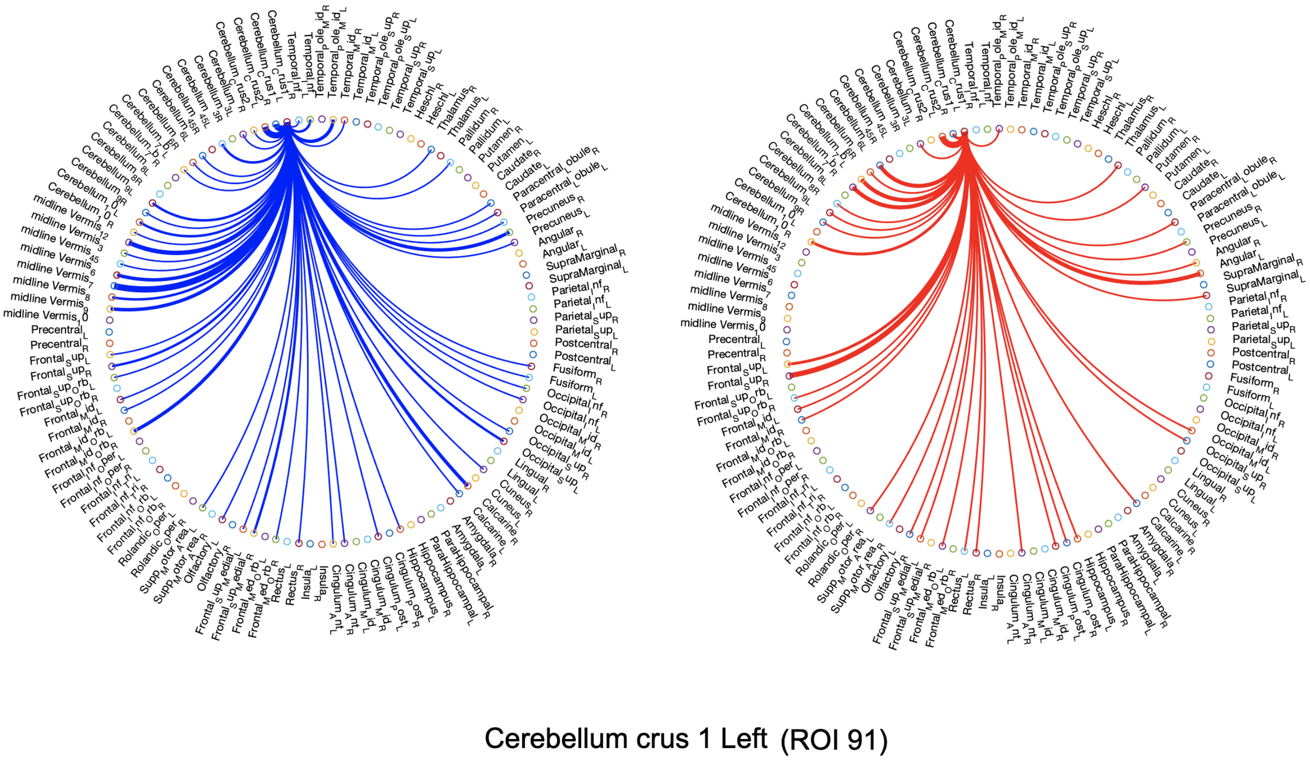

Figure 14. Comparison of average backbone connectivity between left cerebellum crus 1 and other regions between the control and ASD cohort

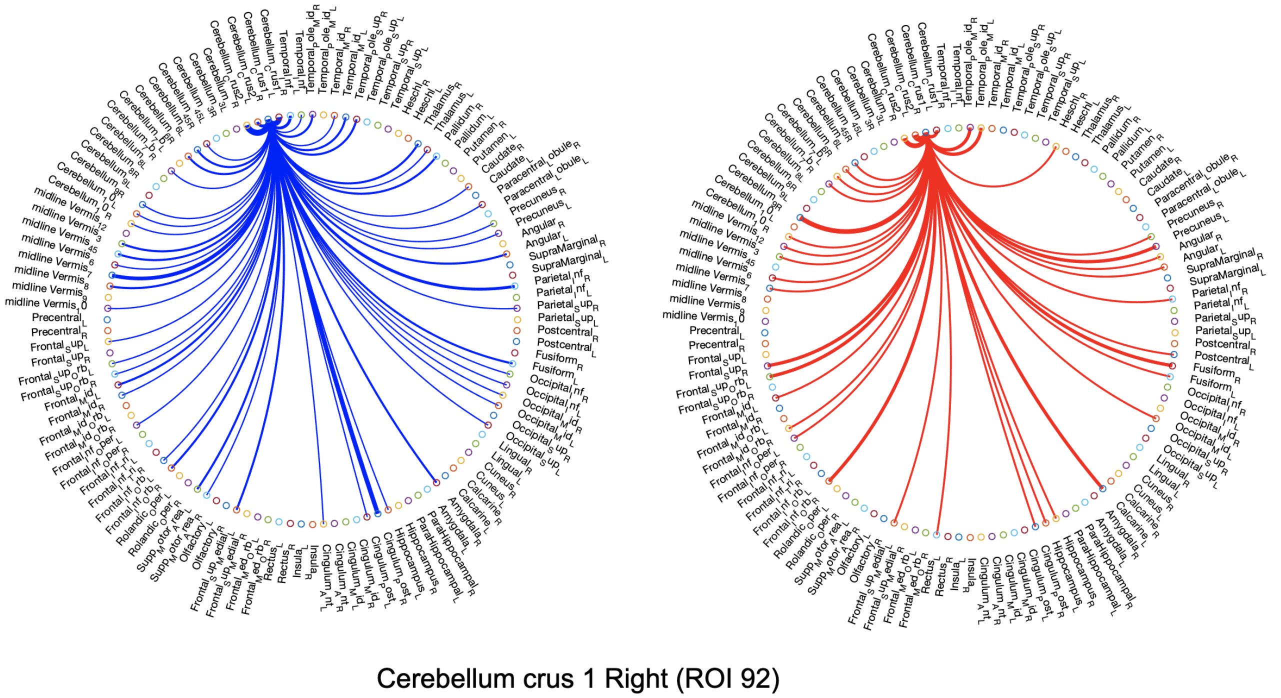

Figure 15. Comparison of average backbone connectivity between right cerebellum crus 1 and other regions between the control and ASD cohort

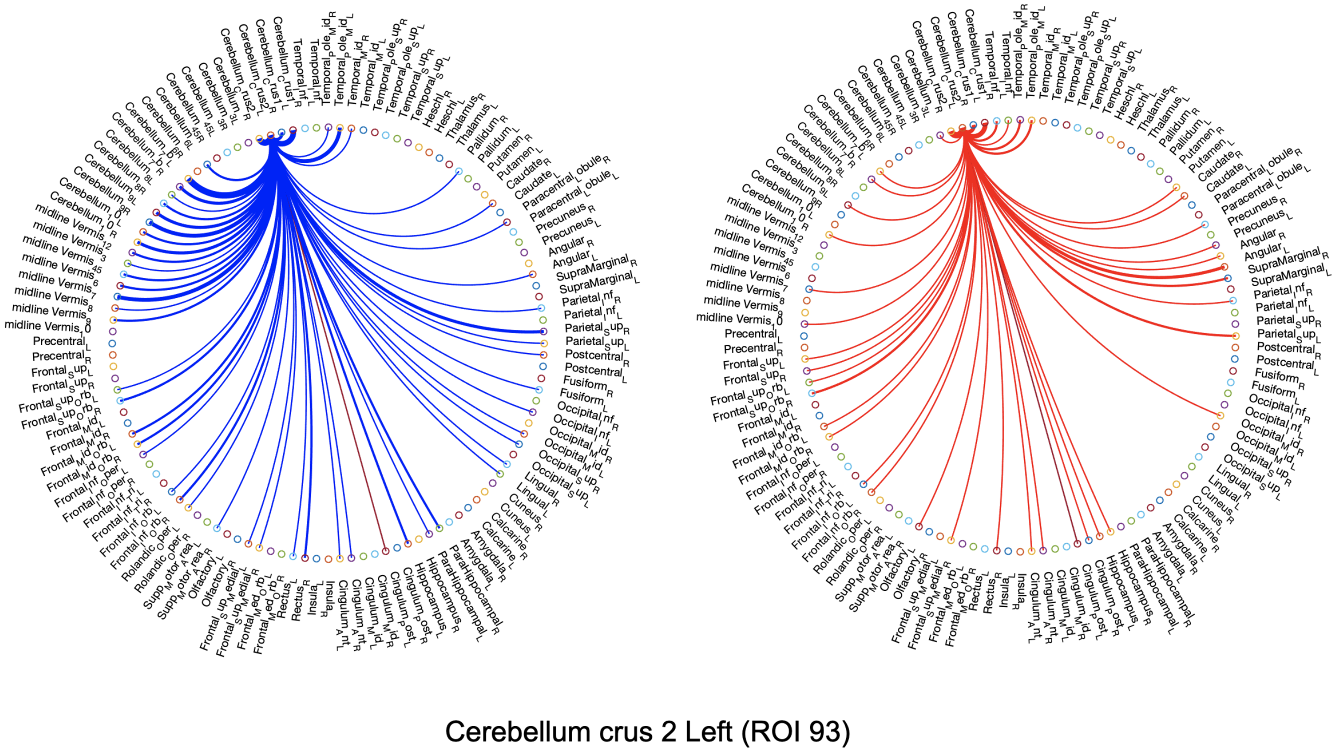

Figure 16. Comparison of average backbone connectivity between left cerebellum crus 2 and other regions between the control and ASD cohort

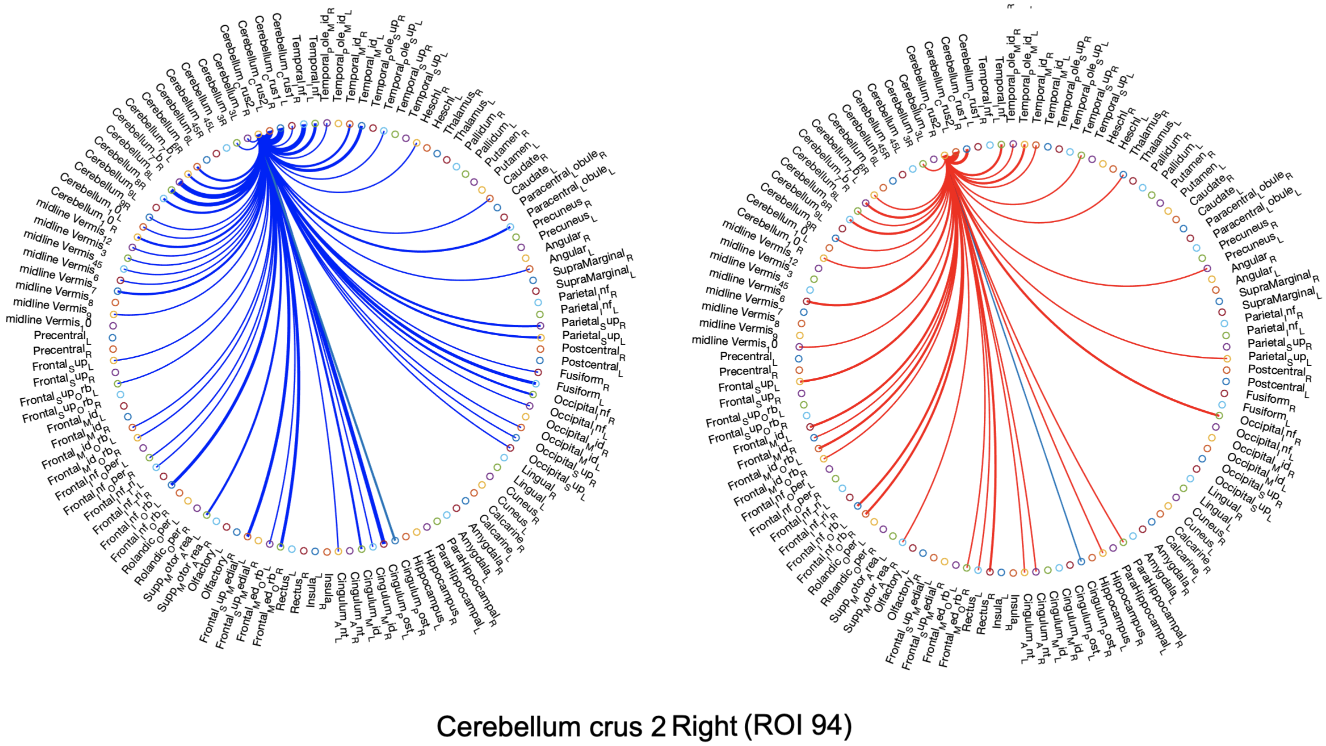

Figure 17. Comparison of average backbone connectivity between right cerebellum crus 2 and other regions between the control and ASD cohort

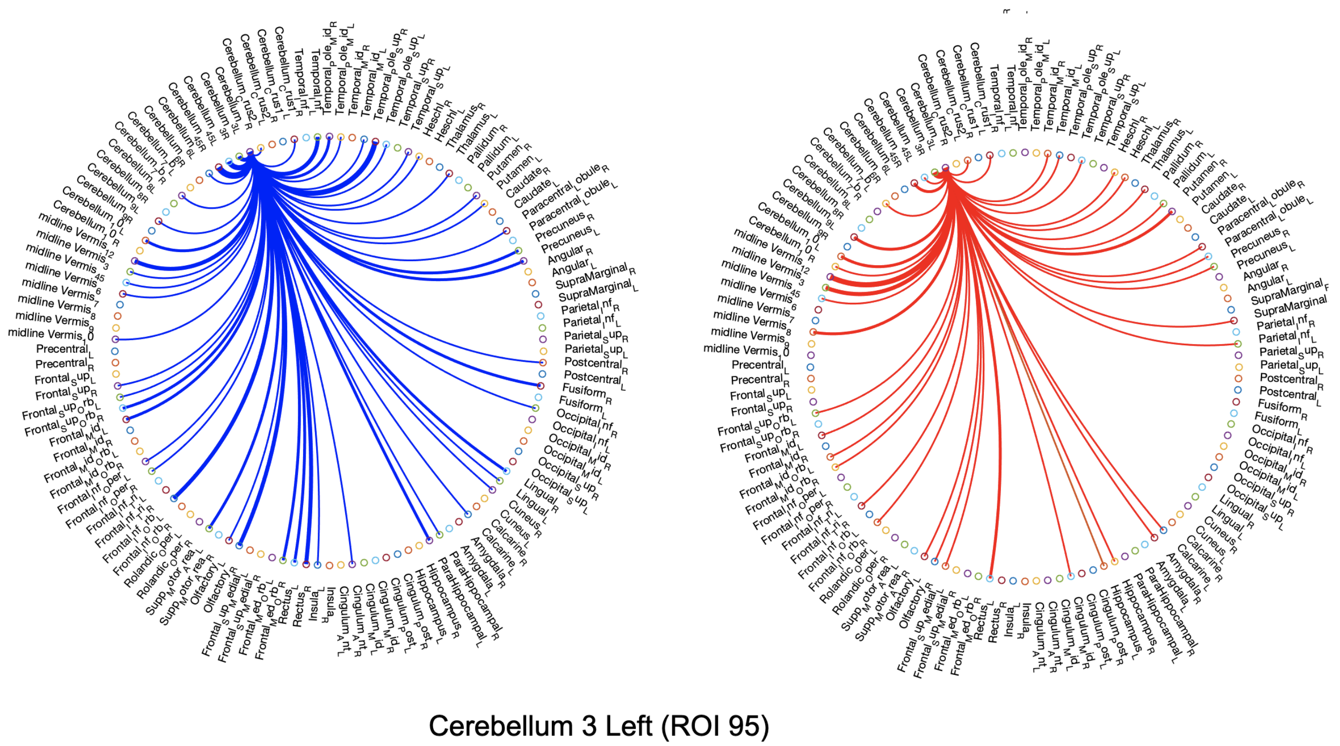

Figure 18. Comparison of average backbone connectivity between left cerebellum area 3 (ROI 95 per AAL) and other regions between the control and ASD cohort

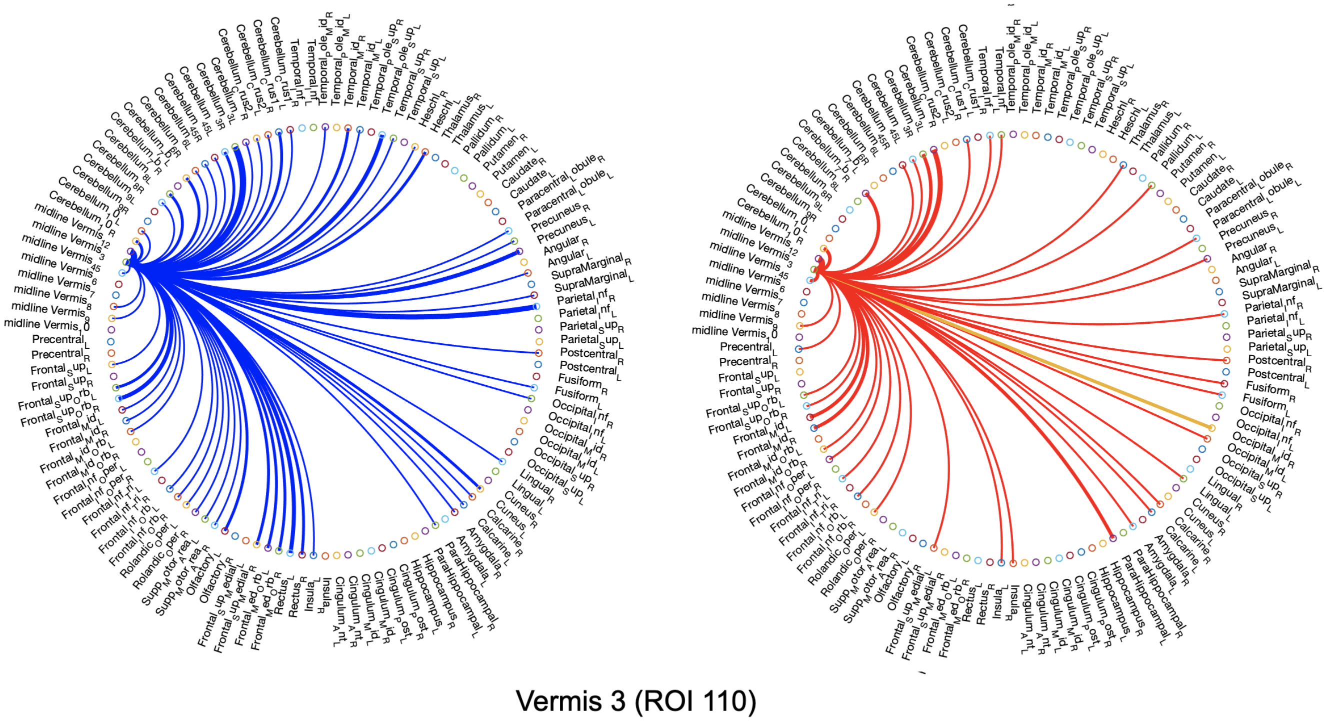

Figure 19. Comparison of average backbone connectivity between the vermis 3 area (ROI 110 per AAL) and other regions between the control and ASD cohort

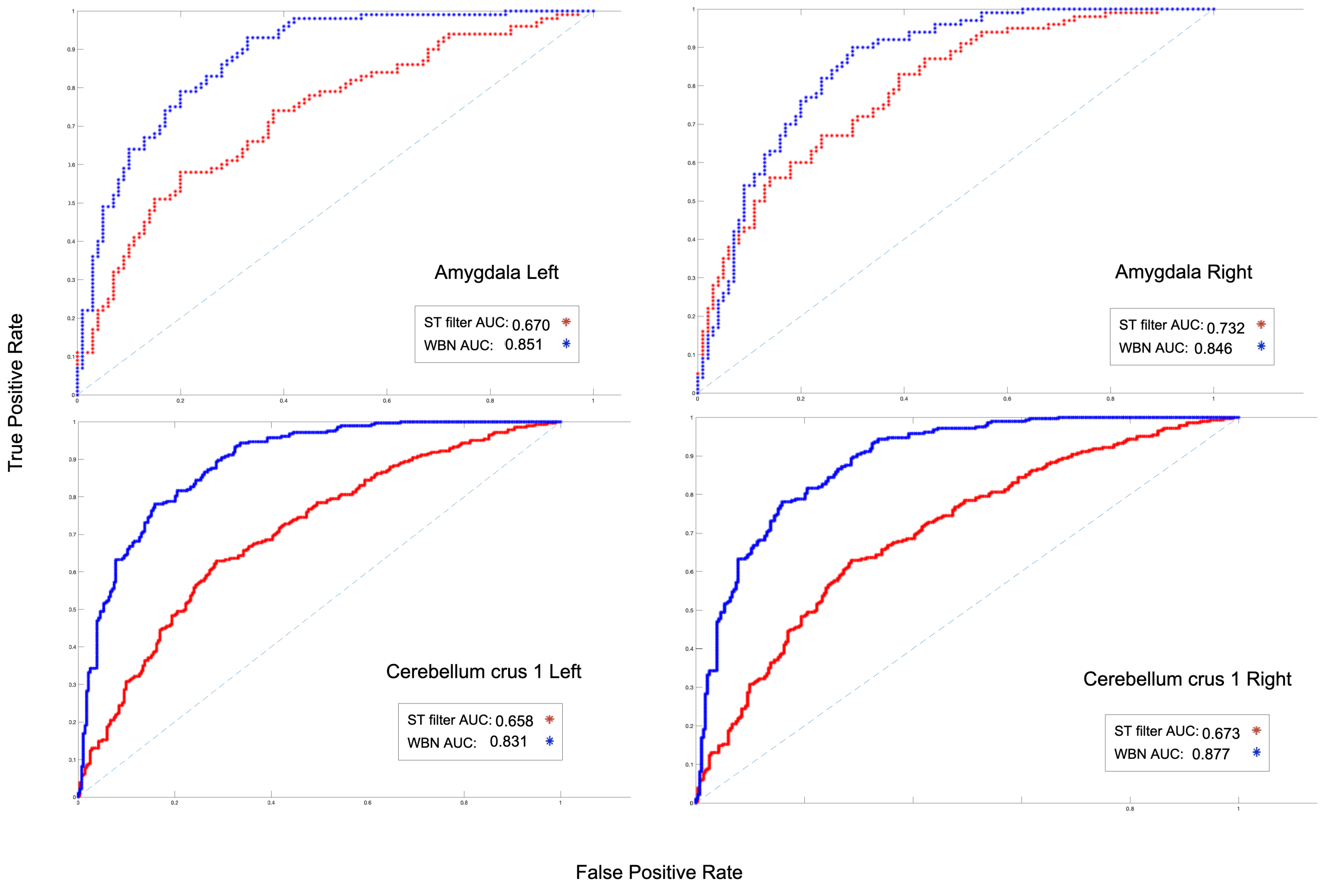

Figure 20. The AUC of detection of injected random weights based on the ST filtering aswell as the proposed approach (WBN) in four different regions where 50 random edges were injected to Amygdalas and 200 random edges were injected to hippocampus areas.
